# Map-based cloning and characterization of *Zea mays male sterility33* (*ZmMs33*) gene, encoding a glycerol-3-phosphate acyltransferase

**DOI:** 10.1007/s00122-018-3083-9

**Published:** 2018-03-15

**Authors:** Ke Xie, Suowei Wu, Ziwen Li, Yan Zhou, Danfeng Zhang, Zhenying Dong, Xueli An, Taotao Zhu, Simiao Zhang, Shuangshuang Liu, Jinping Li, Xiangyuan Wan

**Affiliations:** 1 Advanced Biotechnology and Application Research Center, School of Chemistry and Biological Engineering, University of Science and Technology Beijing, Beijing, 100024 China; 2 Beijing Engineering Laboratory of Main Crop Biotechnology Breeding, Beijing Solidwill Sci-Tech Co. Ltd, Beijing, 100192 China

## Abstract

**Key message:**

Map-based cloning of maize *ms33* gene showed that *ZmMs33* encodes a *sn*-*2* glycerol-3-phosphate acyltransferase, the ortholog of rice OsGPAT3, and it is essential for male fertility in maize.

**Abstract:**

Genetic male sterility has been widely studied for its biological significance and commercial value in hybrid seed production. Although many male-sterile mutants have been identified in maize (*Zea mays* L.), it is likely that most genes that cause male sterility are unknown. Here, we report a recessive genetic male-sterile mutant, *male sterility33* (*ms33*), which displays small, pale yellow anthers, and complete male sterility. Using a map-based cloning approach, maize *GRMZM2G070304* was identified as the *ms33* gene (*ZmMs33*). *ZmMs33* encodes a novel *sn*-*2* glycerol-3-phosphate acyltransferase (GPAT) in maize. A functional complementation experiment showed that *GRMZM2G070304* can rescue the male-sterile phenotype of the *ms33*-*6029* mutant. *GRMZM2G070304* was further confirmed to be the *ms33* gene via targeted knockouts induced by the clustered regularly interspersed short palindromic repeats (CRISPR)/Cas9 system. *ZmMs33* is preferentially expressed in the immature anther from the quartet to early-vacuolate microspore stages and in root tissues at the fifth leaf growth stage. Phylogenetic analysis indicated that ZmMs33 and OsGPAT3 are evolutionarily conserved for anther and pollen development in monocot species. This study reveals that the monocot-specific GPAT3 protein plays an important role in male fertility in maize, and *ZmMs33* and mutants in this gene may have value in maize male-sterile line breeding and hybrid seed production.

**Electronic supplementary material:**

The online version of this article (10.1007/s00122-018-3083-9) contains supplementary material, which is available to authorized users.

## Introduction

Male sterility refers to cases in which viable male gametes (i.e., pollen) are not produced, while female gametes are fully fertile. In maize, there are two general types of male sterility based on the pattern of inheritance: cytoplasmic male sterility (CMS) and genetic male sterility (GMS). CMS is a maternally inherited inability to produce viable pollen that is associated with novel mitochondrial open reading frames (Cui et al. 1996; Feng et al. 2015; Tan et al. 2011). Although a CMS system has been successfully used for maize hybrid seed production, some severe problems, such as the somewhat unsteady heredity of male fertility under certain environments, the narrow germplasm resources of restore lines, the poor genetic diversity between the CMS lines and restore lines, have restricted extensive application in maize (Hu et al. 2006; Kohls et al. 2011; Williams 1995). GMS occurs when lesions in nuclear-encoded genes disrupt normal male gametogenesis. GMS mutants can be either dominant or recessive and typically exhibit Mendelian inheritance (Fox et al. 2017; Tang et al. 2006). The majority of the mutant phenotypes identified to date are controlled by recessive genes, which provide an excellent means of genetic emasculation for hybrid seed production in maize.

GMS is controlled by nuclear genes, mainly those that affect tapetum or microspore development in flowering plants (Jung et al. 2005). Maize is a monoecious crop, which has a separate tassel (male flower) and ear (female flower) on the same plant, making it advantageous for studying anther and pollen development. In maize, anther and pollen development is very complex, with the expression of about 24,000–32,000 genes in a span of nearly 30 days (Ma et al. 2008; Skibbe et al. 2009). Since the first report of a GMS mutant in maize (Eyster 1921), there have been hundreds of maize male-sterile mutants defined from phenotypic scoring (Timofejeva et al. 2013). However, only a handful of these mutants have been characterized cytologically, and even fewer male-sterile genes have been isolated. Most of them are recessive GMS genes, such as *ZmMs7* (Zhang et al. 2018), *Ms8* (Wang et al. 2013), *Ms9* (Albertsen et al. 2016), *Ms22*/*Msca1*(Albertsen et al. 2009), *Ms23* (Nan et al. 2017), *Ms26* (Djukanovic et al. 2013), *Ms32* (Moon et al. 2013), *Ms45* (Cigan et al. 2001), *APV1* (Somaratne et al. 2017), *IPE1* (Chen et al. 2017), *MAC1* (Kelliher and Walbot 2012; Wang et al. 2012) and *OCL4* (Vernoud et al. 2009). To date, *Ms44* has been the only dominant GMS gene identified in maize, and it encodes a lipid transfer protein which is expressed specifically in the tapetum (Fox et al. 2017). The cloning and functional characterization of these male-sterile genes have contributed significantly to our understanding of the molecular mechanisms of anther and pollen development in maize and have provided useful genetic resources for genetic engineering of male-sterile lines for hybrid seed production. Nevertheless, compared with the deep understanding of the molecular mechanisms and gene networks related to anther and pollen development in the model species *Arabidopsis* and rice (Gomez et al. 2015; Shi et al. 2015), comparatively little is known about the equivalent processes in maize.

Glycerol-3-phosphate acyltransferase (GPAT; EC2.3.1.15) is the key enzyme in the glycerolipid synthetic pathway, catalyzing the first dedicated step in the biosynthesis of membrane lipids, storage lipids, and extracellular lipid polyesters (cutin and suberin) (Fig S1). In general, GPATs use acyl-CoA as a substrate, from which they transfer the acyl chain onto glycerol-3-phosphate. The *sn*-*1* GPATs mediate the acylation at the *sn*-*1* position of glycerol-3-phosphate to produce lysophosphatidic acid (LPA). LPA is an important intermediate for the formation of various types of lipids, such as extracellular lipid polyesters, storage and membrane lipids. Some GPATs possess a phosphatase domain that results in *sn*-*2* monoacylglycerol (2-MAG) rather than LPA as the major product. These are named *sn*-*2* GPATs, and they are involved in cutin and suberin synthesis in land plants. To date, most GPATs have been found to be *sn*-*2* GPATs (Beisson et al. 2012; Li et al. 2007; Yang et al. 2012).

In the *Arabidopsis* genome, there are ten GPATs, which can be divided into two families: *sn*-*1* and *sn*-*2* GPAT. The *sn*-*1* GPAT family includes ATS1 and AtGPAT9. ATS1 is a soluble and cytoplasm-localized GPAT that utilizes acyl-ACP substrates and exhibits *sn*-*1* acyl transfer regiospecificity (Nishida et al. 1993). AtGPAT9 is an ER-localized GPAT enzyme responsible for plant membrane lipid and oil biosynthesis in developing *Arabidopsis* seeds and leaves, as well as lipid droplet production in developing pollen grains. The *AtGPAT9* knockout mutant demonstrates both male and female gametophytic lethality phenotypes (Shockey et al. 2016). The *sn*-*2* GPAT family, which consists of the remaining eight members from AtGPAT1 to AtGPAT8 belonging to a land-plant specific family with three distinct clades, are required for the synthesis of cutin and suberin. AtGPAT4/6/8 comprise the cutin-associated clade and are unique bifunctional enzymes with *sn*-*2* acyltransferase and phosphatase activity resulting in 2-MAG products (Yang et al. 2012). The suberin-associated clade includes AtGPAT5/7. AtGPAT5 is an acyltransferase with broad acyl-CoA specificity that is required for root and seed coat suberin biosynthesis. AtGPAT7 exhibits wounding-induced expression, produces suberin-like mononers when overexpressed and likely functions in suberin biosynthesis (Yang et al. 2012). Finally, AtGPAT1/2/3 comprise the third clade. AtGPAT1 possesses *sn*-*2* acyltransferase but not phosphatase activity and can utilize discarboxylic acyl-CoA substrates (Zheng et al. 2003). However, no acyltransferase activity for AtGPAT2 and AtGPAT3 has been detected, and the corresponding mutants display no obvious phenotypes or changes in their polymeric lipids in flowers, leaves or seeds (Yang et al. 2012).

GPATs play important biological roles in plant pollen and anther development. For example, AtGPAT1 is a membrane-bound GPAT protein that was found to be important for tapetum differentiation and nutrient secretion. Consequently, disruption of *AtGPAT1* causes abortion of microspores before maturity. AtGPAT6 plays diverse roles in the development of pollen exine and coat, tapetum formation and stamen elongation. Both *Arabidopsis gpat1* and *gpat6* mutants display altered endoplasmic reticulum (ER) profiles in tapetal cells as well as severely reduced pollen production and decreased pollination (Li et al. 2012; Zheng et al. 2003). Similarly, the *gpat6* mutant showed disturbed pollen formation in tomatoes (Petit et al. 2016). However, the molecular mechanism of GPATs in monocots is largely unknown. GPATs in chloroplasts are responsible for the selective incorporation of saturated and unsaturated fatty-acyl chains into chloroplast membrane, which is an important determinant of a plant’s ability to tolerate chilling temperatures. There was a report on the substrate selectivity and association with chilling tolerance of a rice plastid-localized GPAT (Zhu et al. 2009). Most recently, one member of the rice GPAT family, OsGPAT3, was reported to be involved in anther development and pollen formation (Men et al. 2017). However, none of the *GPAT* genes in maize have been investigated until now.

In this study, the maize *ms33* gene (*ZmMs33*) was isolated by a map-based cloning approach. *ZmMs33* encodes a novel GPAT protein in maize. We characterized *ZmMs33* at multiple levels, including phenotypic observation of *ms33* mutants, gene structure analysis, functional complementation, targeted mutagenesis induced by a CRISPR/Cas9 system, spatio-temporal expression patterns and phylogenetic analysis. *ZmMs33* is the first *GPAT* gene isolated and characterized in maize, and our results will contribute to the understanding of the molecular mechanism of male sterility in maize. More importantly, *ZmMs33* and mutants in this gene may have value in maize male-sterile line breeding and hybrid seed production.

## Materials and methods

### Plant materials and growth conditions

*ms33*-*6019* (Stock ID: 228F), *ms33*-*6029* (Stock ID: 228H), *ms33*-*6038* (Stock ID: 228I) and *ms33*-*6052* (Stock ID: 206F) mutants were obtained from the Maize Genetics Cooperation Stock Centre (http://maizecoop.cropsci.uiuc.edu). The two F_2_ mapping populations were derived from crosses of *ms33*-*6029 *× Chang7-2 and *ms33*-*6038 *× Chang7-2. All the plants were grown in the field in Beijing or Sanya, China. The T_0_ transgenic plants and their progeny were grown in a greenhouse in Beijing, China.

### Characterization of mutant phenotypes

Based on the methods as described previously (Zhang et al. 2018), tassels and spikelets were photographed, pollen grains were stained with 1% I_2_-KI and photographed. Anther staging was defined as described by Zhang et al. (2018). SEM of anthers at stage 13 was performed as described by Chen et al. (2017). Anther staging was defined as described by Albertsen and Phillips (1981).

### Map-based cloning

Genomic DNA was extracted from maize leaves using the CTAB method with some modifications (Wan et al. 2006). As the *ms33* locus was mapped to the long arm of maize chromosome 2 (2L) by using B-A translocations (Patterson 1995) and RFLP markers (Trimnell et al. 1999), nine SSR primer pairs on maize chromosome 2L were chosen for *ms33* primary mapping (Table S1). For fine mapping, several InDel markers (Table S1) were designed with DNAMAN6.0 (LynnonBiosoft). All PCR primers for these markers were synthesized by Sangon Biotech (Shanghai, China) and tested to identify polymorphic markers distinguishing fertile from sterile plants. By scoring the presence/absence of recombinants at diverse marker locations (Wan et al. 2008), the *ms33* gene was narrowed down to a 349-kb interval on chromosome 2L.

### Plasmid construction for functional complementation and CRISPR/Cas9 mutagenesis

For function complementation, the *ZmMs33* gene native promoter (1794 bp, Fig. S5) was amplified from maize B73 using primer pair Ms33-ProP, and the *ZmMs33* coding DNA sequence (1578 bp, Figs. S6 and S7) was amplified from the cDNA of B73 anthers using primer pair Ms33-CDSP. The two fragments were fused together with *Hin*dIII/*Bam*HI and then subcloned into *pBCXUN* (Chen et al. 2009) with the hygromycin coding region replaced by an herbicide resistance marker (*Bar*). The resultant vector was named *pZmMs33pro::ZmMs33*. For site-directed mutagenesis of *ZmMs33*, three types of CRISPR/Cas9 plasmids were constructed based on the pBUE411 vector as described previously (Xing et al. 2014). For assembly of one gRNA, the double-stranded short DNA was annealed from two complementary oligos of Cas9-1g-1 (Table S2) and inserted into the pBUE411 vector by *Bsa*I digestion. The colonies were confirmed by sequencing and the corresponding vector was named as pCas9-1g (Fig. 6). For assembly of two gRNAs, PCR fragments were amplified from pCBC-MT1T2 with primers sets (Table S2) among which target sites were incorporated, and then, the purified PCR fragments were digested with *Bsa*I and cloned into the pBUE411 vector. The colonies were confirmed by sequencing and the corresponding vectors were designated as pCas9-2g-1 or pCas9-2g-2 (Fig. 6).

### Maize genetic transformation

All constructs were transformed into the maize Hi-II hybrid line using the method as described previously (Zhang et al. 2018). The *Bar* gene was employed as a selectable marker and the transformants were screened by PCR amplification using primer pair Bar-P. The transgenic T-DNA region was then transferred into the *ms33*-*6029* mutant by crossing and backcrossing. To confirm the presence of the *ms33*-*6029* allele and the transgenic T-DNA region in the progeny, the transgenic plants were screened by PCR amplification using primer pairs ms33-ID and Bar-P. All primers described above are listed in Table S2.

### Protein alignment and phylogenetic analysis

Sixty homologues of ZmMs33 were obtained by a Basic Local Alignment Searching Tool-protein to protein (BLAST_P_) search on the National Center for Biotechnology Information (NCBI), Rice Genome Annotation Project (RGAP) and Maize Genetics and Genomics Database (MaizeGDB) websites. The phylogenetic tree was generated in MEGA7.0 using the maximum likelihood method (Kumar et al. 2016). The amino acid sequences of maize ZmMs33 and the orthologous proteins in barley, rice, sorghum and millet were aligned using DNAMAN6.0, and the conserved domains were analysed with a Conserved Domain Search Service (CD Search) in the NCBI website.

### RNA extraction and expression analysis

Total maize RNA was isolated using the TRIzol reagent (Invitrogen) from the following maize tissues: anthers during different stages, leaves, roots and immature ears. Total RNA (1 µg) was used to synthesize the first-strand cDNA with Superscript III RT (Invitrogen). Semi-quantitative reverse-transcription PCR (RT-PCR) analyses were conducted using 1 µL cDNA as template. RT-PCR was performed on a Bio-Rad MyCycler Thermal Cycler with a standard three-step protocol, consisting of 94 °C for 2 min followed by 30 cycles of 94 °C for 30 s, 58 °C for 30 s and 72 °C for 30 s. *ZmActin1* and *GAPDH* were used as the internal controls. All primers used for RT-PCR are listed in Table S2.

*ZmMs33* GenBank accession number: MF615247.

## Results

### Maize *ms33* is a single recessive mutant exhibiting complete male sterility

**Fig. 1:**
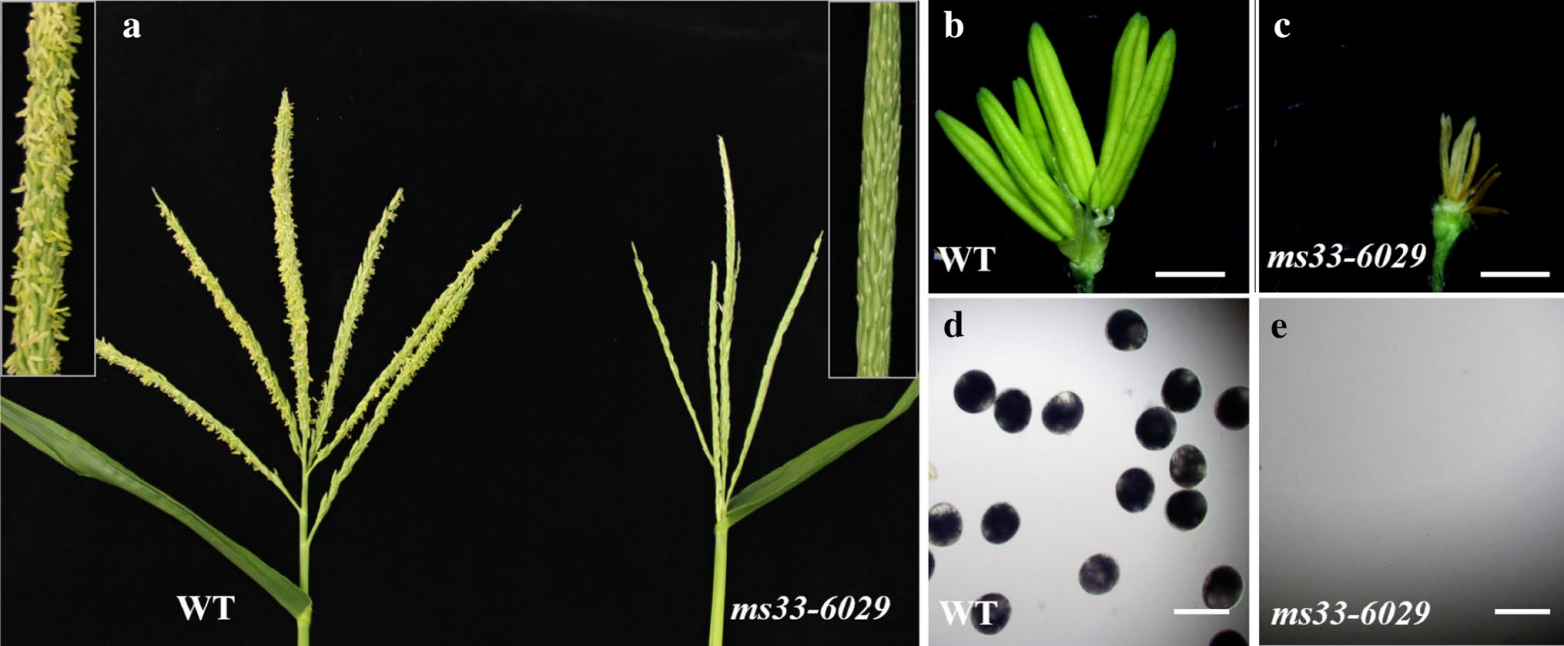
Phenotypic comparison of the wild type (WT) and *ms33*-*6029* mutant. **a** The tassels of WT (left) and *ms33*-*6029* (right). **b**, **c** The spikelet of WT and *ms33*-*6029* with the glume, lemma and palea removed. **d**, **e** The pollen of WT and *ms33*-*6029* stained with I_2_-KI. Bars = 2 mm (**b**, **c**), 100 μm (**d**, **e**)

Two *ms33* mutants, *ms33*-*6029* and *ms33*-*6038*, were obtained from the Maize Genetics Cooperation Stock Centre. Compared with the wild-type male-fertile sibling, *ms33*-*6029* displayed complete male sterility with no exerted anthers (Fig. 1a) but normal vegetative growth and female fertility. The mutant anthers were thin and pale yellow (Fig. 1b, c) and lacked pollen grains in the withered anthers, which were not stained with I_2_-KI (Fig. 1d, e). The mutant phenotype was stable under multiple environments, including different years (2013–2016) and locations (Beijing or Sanya, China, data not shown) (Patterson 1995; Trimnell et al. 1999). The *ms33*-*6038* mutant also showed a similar male-sterile phenotype as the *ms33*-*6029* mutant (Fig. S2).

The two *ms33* mutants were crossed with the maize inbred line Chang7-2. All the F_1_ progeny was male fertile, and the F_2_ population segregated at a male fertile-to-sterile ratio of 3:1 (Table 1), suggesting that both *ms33*-*6029* and *ms33*-*6038* were single recessive mutations. Furthermore, when the *ms33*-*6029/ms33*-*6029* homozygous plants were outcrossed using pollen from *ms33*-*6038/*+ heterozygous plants, the F_1_ progeny showed a 1:1 (101:98) segregation ratio of male sterile to fertile plants, proving that the mutant gene of *ms33*-*6029* is allelic to that of *ms33*-*6038*.

**Table 1:** The ratio of fertile to sterile plants in the two F_2_ populations of the *ms33* mutant

F_2_ population	Total plants	Fertile plants, *F*	Sterile plants, *S*	*F*/*S* ratio	*χ* ^2^	*P*	Significant test, *P* > 0.05
*ms33*-*6029 * × Chang7-2	916	681	235	2.9:1	0.087	0.7681	ns*
*ms33*-*6038 *× Chang7-2	966	712	254	2.8:1	0.3435	0.5578	ns*

ns* means non-significant

### Anther and tapetum development was defective in the *ms33* mutant

**Fig. 2:**
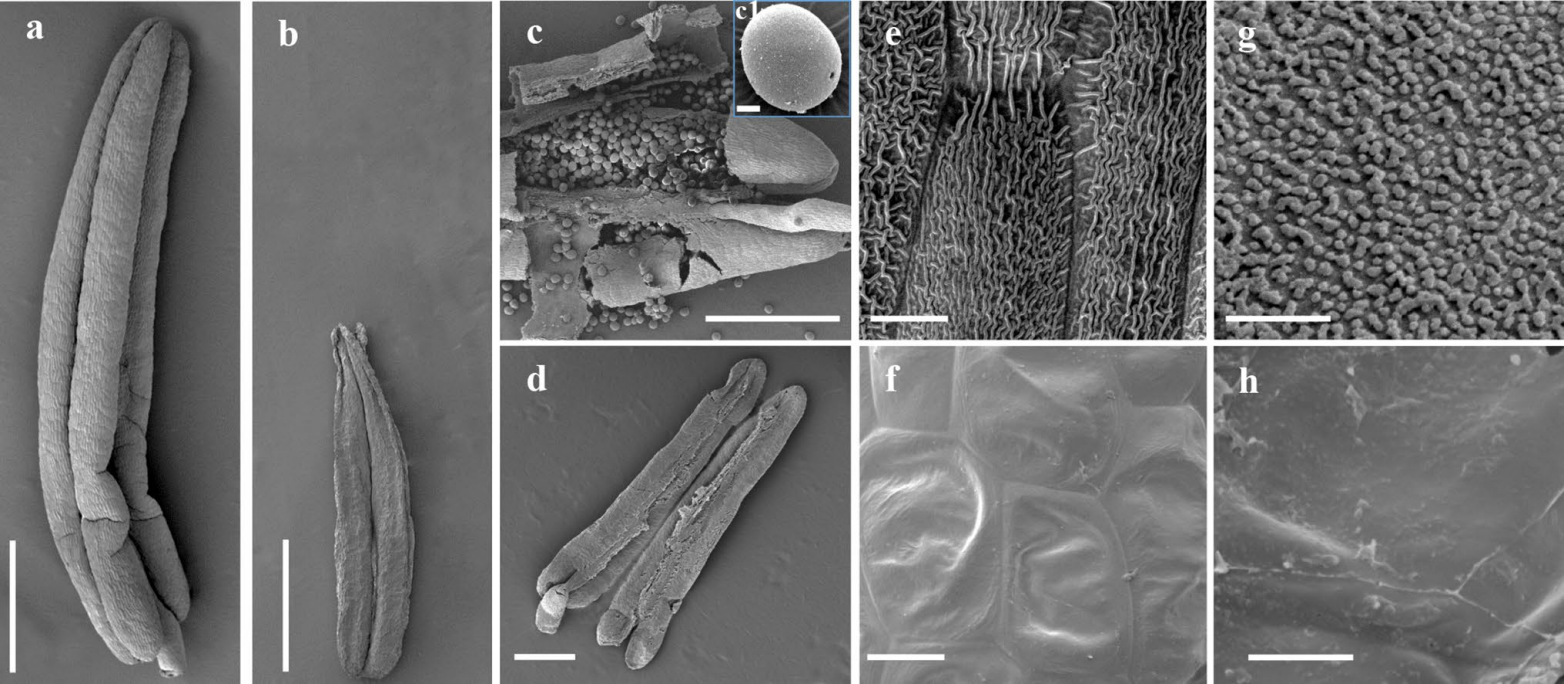
Appearance of the anther and pollen grain in the wild type (WT) and *ms33*-*6029* mutant at stage 13 of anther development under scanning electron microscopy. **a** WT and **b** *ms33*-*6029* anthers. **c**, **c1**, **d**, Pollen grains of WT (**c**, **c1**) and the absence of pollen grains in *ms33*-*6029* (**d**). **e**, **f** The outermost surface of the epidermis of WT (**e**) and *ms33*-*6029* (**f**) anthers. **g**, **h** The inner surface of the anther wall layers of WT (**g**) and *ms33*-*6029* (**h**) anthers. Bars = 1 mm (**a**, **b**), 500 μm (**c**, **d**), 15 μm (**c1**), 10 μm (**e**, **f**), 5 μm (**g**, **h**)

Scanning electron microscopy (SEM) was used to observe phenotypic differences between the *ms33* mutants and wild type at the mature pollen grain stage. Consistent with the above morphological results, the *ms33*-*6029* mutant produced small, wilted anthers with no pollen grains in the anther locule (Fig. 2a–d). A reticulate cuticle coated the outermost surface of anthers, and abundant Ubisch bodies were distributed on the outside of tapetal cells in wild-type anthers (Fig. 2e, g). However, the *ms33*-*6029* anther surface was shrunken and without a reticulate coat (Fig. 2f), indicating that anther cuticle formation was disrupted in the mutant. In addition, Ubisch bodies were not observed on the inner surface of mutant anthers (Fig. 2h). The *ms33*-*6038* mutant also showed similar anther developmental defects at the same stage (Fig. S3). These results showed that *ms33* mutation influences development of the anther cuticle and Ubisch bodies, as well as the formation of pollen grains.

### Fine mapping of the *ms33* male-sterility gene

**Fig. 3:**
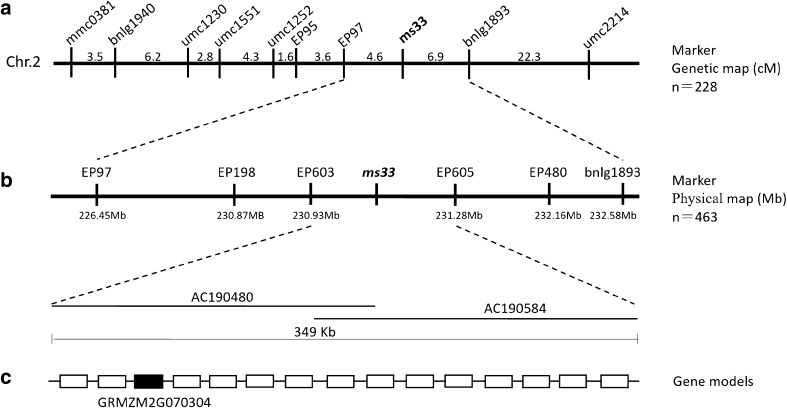
Map-based cloning of the maize *ms33* gene. **a** Primary mapping of the *ms33* gene between markers EP97 and bnlg1893. **b** Fine mapping of the *ms33* gene to an interval of nearly 349 kb between markers EP603 and EP605. **c** The 15 putative gene models in the interval. Among them, *GRMZM2G070304*, similar to the rice *OsGPAT3* gene, is the candidate gene. *n* the number of the male-sterile plants used in the F_2_ mapping population is showed on the right

*ms33* was mapped to the long arm of chromosome 2 by B-A translocation and RFLP markers (Patterson 1995; Trimnell et al. 1999). A map-based cloning approach was adopted to isolate the *ms33* mutant gene. Using 228 male-sterile individuals from the F_2_ population and molecular marker linkage analysis, the *ms33* locus was initially mapped between SSR markers EP97 and bnlg1893 on chromosome 2L (Fig. 3a). For fine mapping, four InDel markers in the interval were developed (Table S1) and *ms33* was narrowed down to a 349-kb interval between the InDel markers EP603 and EP605 (Fig. 3b). According to the annotation from MaizeGDB, fifteen gene models are predicted in this region (Fig. 3c and Table S3), including *GRMZM2G070304*, is likely to be the *ms33* gene based on bioinformatic analysis and sequence comparison between the wild type and two *ms33* mutants (see below).

### Map-based cloning and functional confirmation of *ms33* gene in maize

**Fig. 4:**
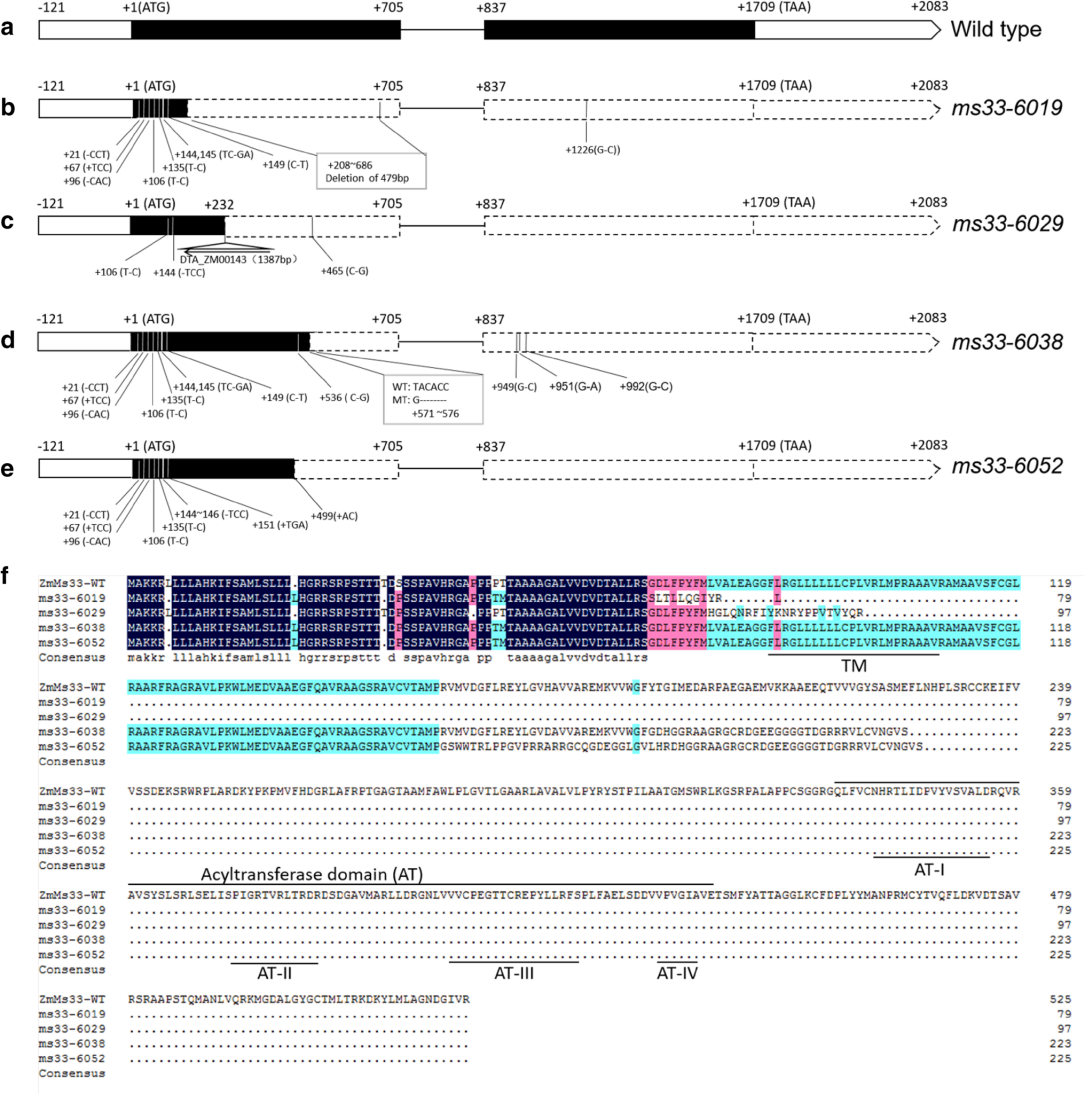
The gene structure and predicted amino acid sequences of the maize *ZmMs33* candidate gene (*GRMZM2G070304*). **a** A schematic representation of two exons and one intron of *ZmMs33* in wild type. + 1 indicates the putative starting nucleotide of translation, and the stop codon (TAA) is + 1709 in wild type. Black boxes indicate exons, and intervening lines indicate introns. **b** In *ms33*-*6019*, besides the 479-bp deletion at the + 208–686, there are three InDels and five SNPs in Exon 1 and one SNP in Exon 2. **c** In *ms33*-*6029*, besides the transposon insertion at the + 232 site, there are two SNPs and a 3-bp deletion. **d** In *ms33*-*6038*, besides the 5-bp deletion at the + 572–576, there are three InDels and seven SNPs in Exon 1 and three SNPs in Exon 2. **e** In *ms33*-*6052*, besides the 2-bp insertion at the + 499–500, there are five InDels and two SNPs in Exon 1. **f** The insertion or deletion mutations in *ms33*-*6019*, *ms33*-*6029*, *ms33*-*6038 a*nd *ms33*-*6052* caused frame-shift mutations and altered the reading frame after the 69th, 77th, 188th and 160th amino acid, respectively, resulting in a lack of the conserved acyltransferase domain (AT), which contains four conserved motifs. The putative transmembrane domain (TM) and the conserved AT domain are underlined

*GRMZM2G070304* was identified to have two exons and one intron (Fig. 4a) and was likely to encode a putative 525-amino acid GPAT protein, containing a transmembrane (TM) domain and an acyltransferase (AT) domain (Fig. 4f). Sequencing of this gene in the *ms33*-*6019* mutant revealed that a 479-bp deletion at the + 208–686 nucleotide site in the first exon (Fig. 4b), which caused a frame-shift mutation and altered the open reading frame after the 69th amino acid, resulting in a lack of the conserved AT domain (Fig. 4f). Additionally, there were three InDels and five SNPs in the first exon and one SNP in the second exon (Fig. 4b), which resulted in deletion of two amino acids, one amino-acid insertion, and four amino-acid changes (Fig. 4f). Sequencing of this gene in the *ms33*-*6029* mutant revealed that a 1387-bp transposable element (DTA_ZM00143) was inserted at the + 232 nucleotide site in the first exon (Fig. 4c), which caused a frame-shift mutation and altered the open reading frame after the 77th amino acid, resulting in a lack of the conserved AT domain (Fig. 4f). Additionally, there were two SNPs and a 3-bp deletion in the first exon (Fig. 3c), leading to a single amino-acid change and deletion, respectively (Fig. 4f). Sequencing of this gene in the *ms33*-*6038* mutant revealed a 5-bp (ACACC) deletion at the + 572–576 nucleotide site in the first exon (Fig. 4d), which also caused an in-frame premature stop codon and led to deletion of the conserved AT domain (Fig. 4f). Sequencing of this gene in the *ms33*-*6052* mutant revealed a 2-bp (AC) insertion at the + 499 nucleotide site in the first exon (Fig. 4e), which also caused an in-frame premature stop codon and led to deletion of the conserved AT domain (Fig. 4f). Similar with the *ms33*-*6019*, there were several InDels and SNPs in the first and second exon of *GRMZM2G070304* in the *ms33*-*6038* and *ms33*-*6052* mutants (Fig. 4d, e), which also resulted in deletion of two amino acids, one amino-acid insertion, and four amino-acid changes (Fig. 4f).

**Fig. 5:**
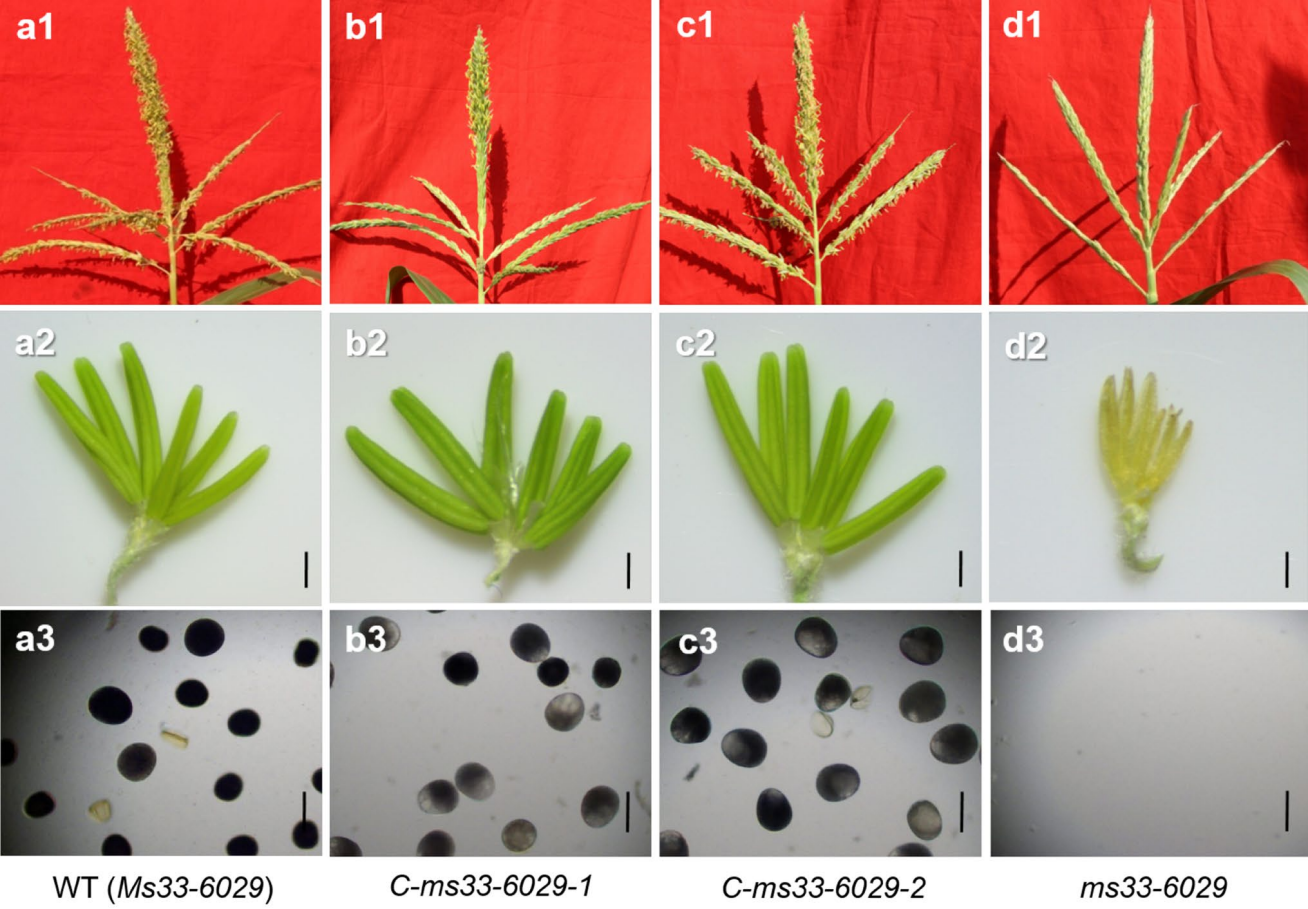
Functional complementation of the maize *ms33*-*6029* mutant. **a1**–**d1** Phenotypes of tassels, **a2**–**d2** spikelets and **a3**–**d3** pollen grains stained with I_2_-KI in **a1**–**a3** the wild type, **d1**–**d3** *ms33*-*6029* mutant and two complemented transgenic lines **b1**–**b3** *C*-*ms33*-*6029*-*1* (*pMs33::Ms33/ms33*-*6029*) and **c1**–**c3** *C*-*ms33*-*6029*-*2* (*pMs33::Ms33/ms33*-*6029*), respectively. Bars = 1 mm (**a2**–**d2**), 100 μm (**a3**–**d3**)

**Fig. 6:**
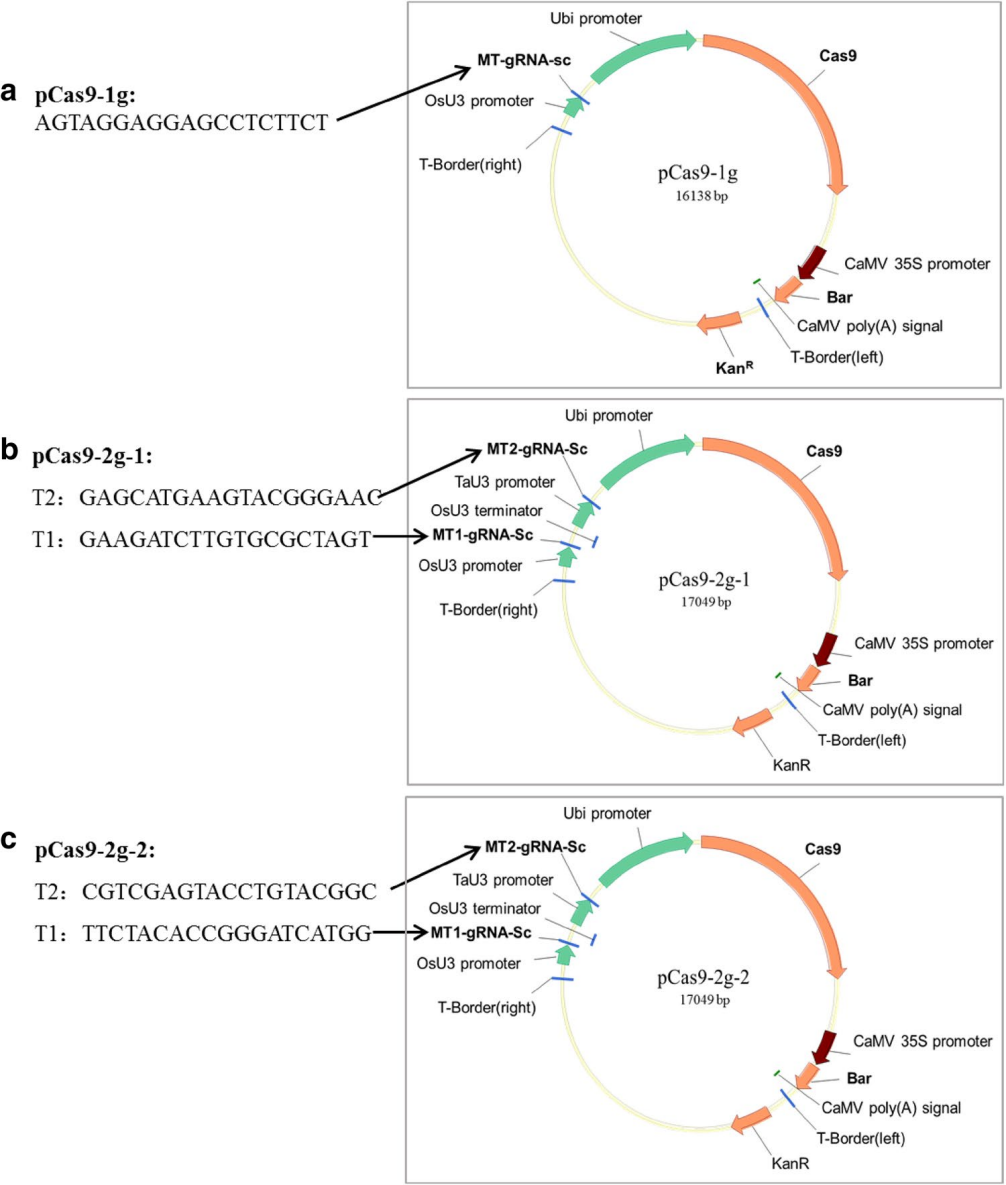
Physical maps of three CRISPR/Cas9 vectors carrying **a** one-gRNA, **b**, **c** two-gRNAs and their corresponding target sites in the *ZmMs33* gene

**Fig. 7:**
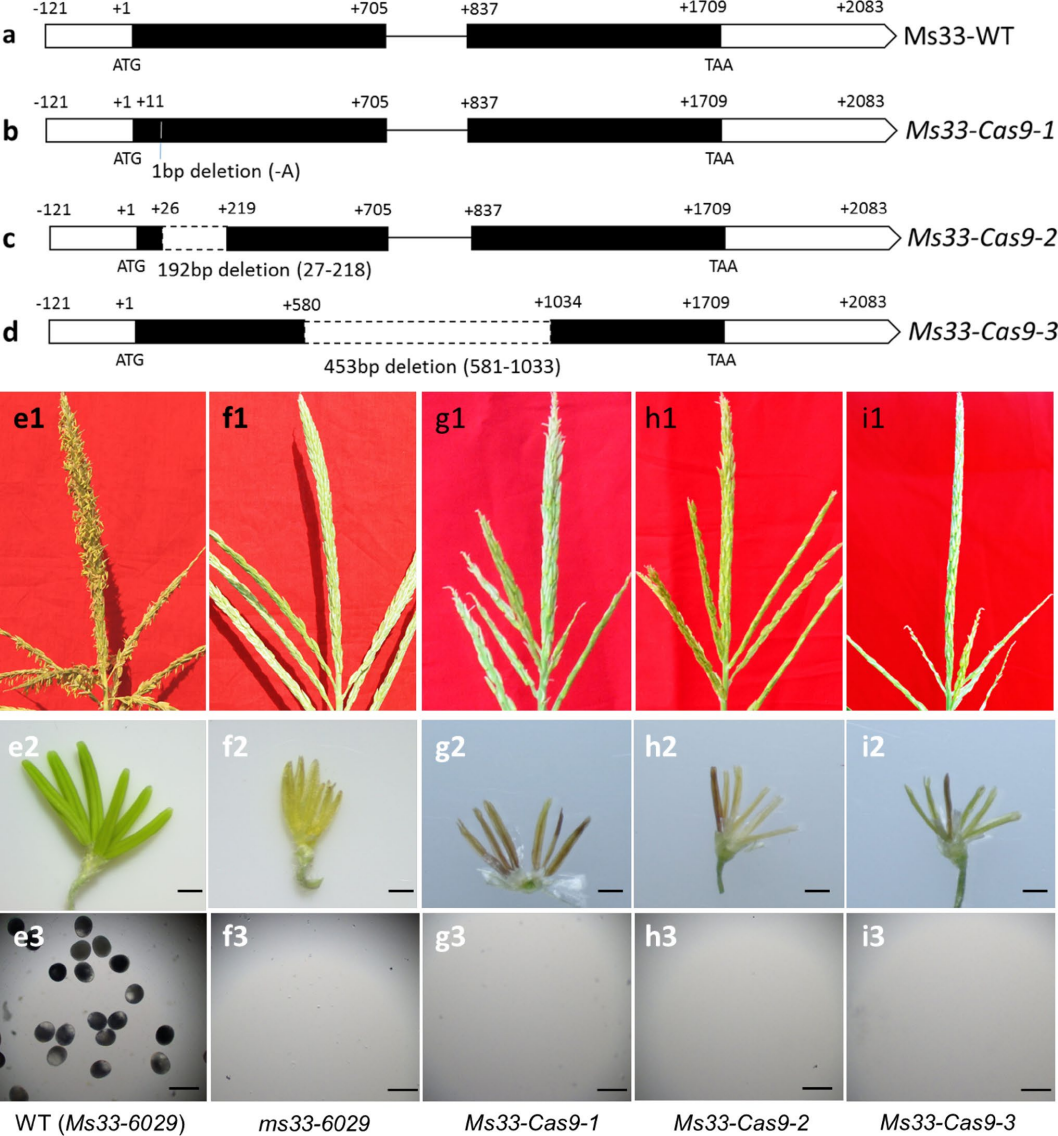
Phenotypes of tassels, spikelets and pollen grains in three knockout lines generated by a CRISPR/Cas9 system. Compared with the wild-type **(a)** sequence, deletions of 1, 192, and 453 bp were detected in the three knockout lines *Ms33*-*Cas9*-*1* **(b)**, *Ms33*-*Cas9*-*2* **(c)**, and *Ms33*-*Cas9*-*3* **(d)**, respectively. **e1** Wild-type tassel, **e2** spikelet and **e3** mature pollen grains stained with 1% I2-KI solution. **f2** The *ms33*-*6029* mutant anthers were not exserted from **f1** the tassel, and **f3** no pollen grains were present in the anthers. **g1**–**i1** The tassels, **g2**–**i2** spikelets and **g3**–**i3** absence of pollen grains in the three knockout lines (*Ms33*-*Cas9*-*1 *~* 3*). Bars = 1 mm (**e2**–**i2**), 150 μm (**e3**–**i3**)

By using *Agrobacterium*-mediated maize transformation, 15 transgenic lines with the coding sequence of *GRMZM2G070304* driven by its own promoter (1.8 kb) restored male fertility in maize *ms33*-*6029* mutant (Fig. 5; Table S4). Furthermore, the *GRMZM2G070304* gene was further confirmed as the *ms33* gene by targeted knockouts using a CRISPR/Cas9 system (Figs. 6, 7). The maize Hi-II hybrid line was transformed with three vectors (1 single gRNA/Cas9 vector and 2 double gRNA/Cas9 vectors; Fig. 6) targeting different sites of endogenous *ZmMs33*. Site-directed mutations were observed at all targeted sites by DNA sequencing analysis in more than 30 independent transgenic events (Fig. S8 and Table S5). The three types of T_0_-generation maize plants homozygous for null alleles of *ZmMs33* were observed to be frame-shift mutations by 1-, 192- and 453-bp deletions (Fig. 7). These results showed that *GRMZM2G070304* is responsible for restoring the male-sterile phenotype in the *ms33* mutants; hereafter, we refer to *GRMZM2G070304* as *ZmMs33*.

### *ZmMs33* is mainly expressed in the anther and root tissues

**Fig. 8:**
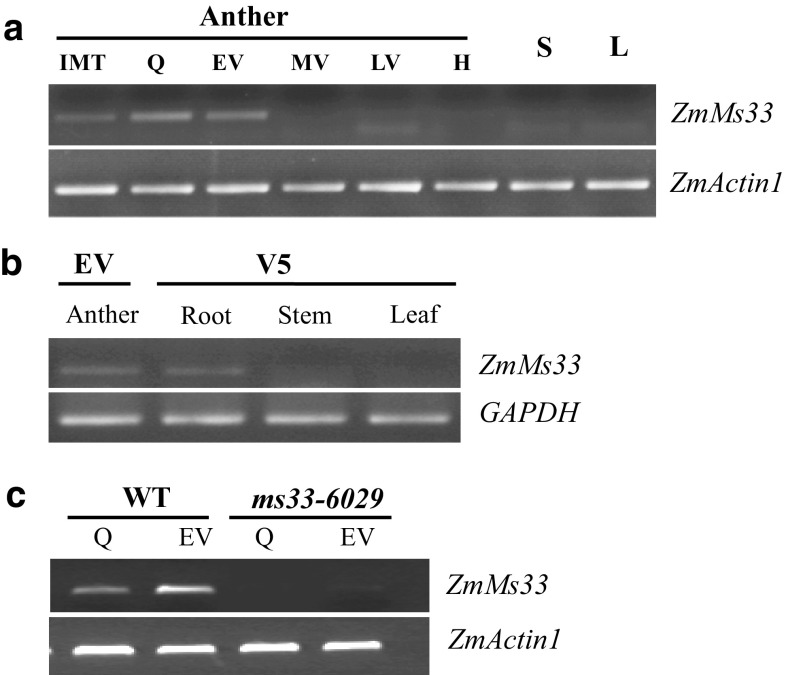
Expression patterns of the maize *ZmMs33* gene by RT-PCR analysis. **a** Maximum expression was observed in the anther during the meiosis quartet to early vacuolate microspore stages. **b** *ZmMs33* expression in maize root at the fifth leaf growth stage, but no expression was detected in maize stem and leaf. **c** Comparison of *ZmMs33* expression in the anther of wild type and the *ms33*-*6029* mutant at the meiosis quartet and early vacuolate microspore stages. *ZmMs33* expression data were normalized against *ZmActin1* or *GAPDH*. Different anther developmental stages are shown: *IMT* immature tassels, *Q* meiosis quartets, *EV* early vacuolate microspore, *MV* middle vacuolate microspore, *LV* late vacuolate microspore, *H* heading, *S* stem, *L* leaf, *V5* the fifth leaf growth stage

To analyse the expression pattern of *ZmMs33*, semi-quantitative RT-PCR was performed. The results revealed that *ZmMs33* was mainly expressed in premature anthers from the quartet to early-vacuolate microspore stages and in root tissues at the fifth leaf growth stage (Fig. 8a, b). Compared to the wild type, the expression of *ZmMs33* was hardly detected in the premature anthers of the *ms33*-*6029* mutant (Fig. 8c). The anther-preferential expression pattern of *ZmMs33* is consistent with its function in anther development. Although relatively higher levels of *ZmMs33* expression were also detected in root tissues during the fifth leaf growth stage, and further confirmed to be *ZmMs33* cDNA by sequencing the RT-PCR products (Fig. S4), no obvious morphological abnormalities were observed in the roots of *ms33*, suggesting a possible redundant function of *ZmMs33*-related homologues in the root tissues.

### ZmMs33 is the orthologue of OsGPAT3, belonging to a monocot-specific *sn*-*2* GPAT family

**Fig. 9:**
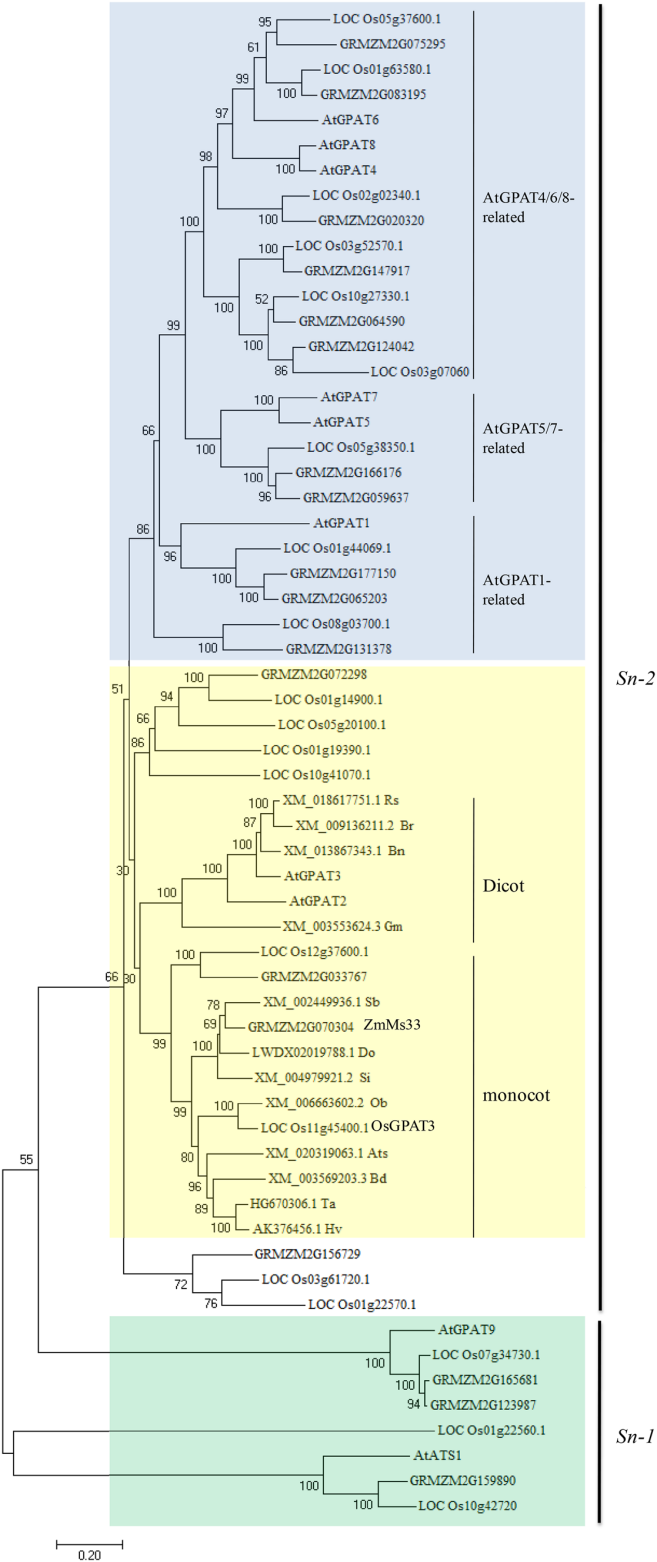
Phylogenetic analysis of ZmMs33 and related proteins. The evolutionary analyses were conducted in MEGA7 using the maximum likelihood method based on the Poisson correction model. The tree with the greatest log likelihood is shown. The analyses involved 48 amino acid sequences from *Zea mays* (Zm), *Oryza sativa* (Os) and *Arabidopsis thaliana* (At), and 12 putative GPATs from other plants, including *Oryza brachyantha* (Ob), *Sorghum bicolor* (Sb), *Triticum aestivum* (Ta), *Bradchypodium distachyon* (Bd), *Aegilops tauschii (*Ats), *Dichanthelium oligosanthes* (Do), *Hordeum vulgare* (Hv), *Setaria italica* (Si), *Glycine max* (Gm), *Brassica napus* (Bn), *Raphanus sativus* (Rs) and *Brassica rapa* (Br)

**Fig. 10:**
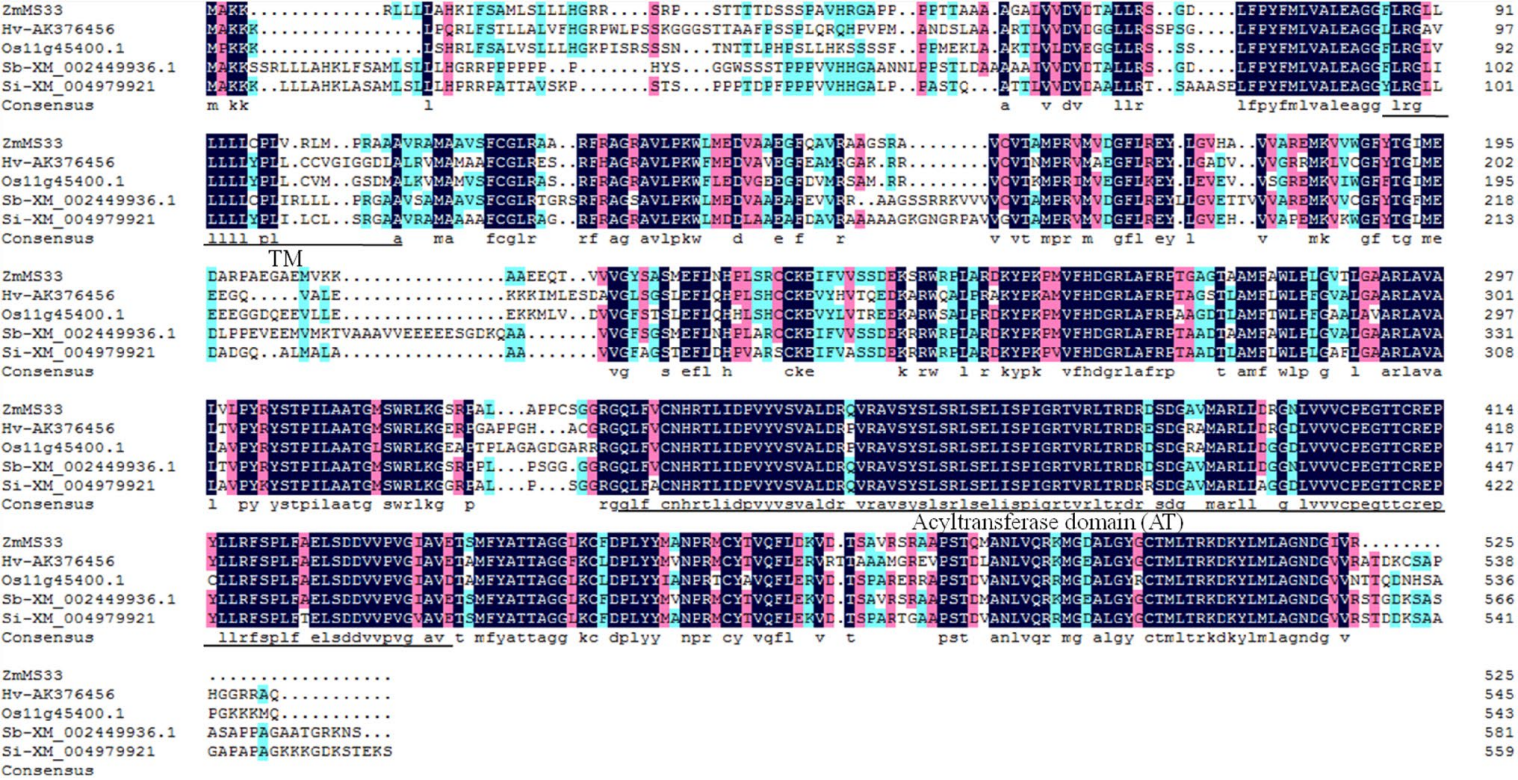
The amino acid sequence alignment of maize ZmMs33 and orthologous proteins. Hv-AK376456 (*Hordeum vulgare*), Os11g45400.1 (OsGPAT3, *Oryza sativa*), Sb-XM_002449936.1 (*Sorghum bicolor*), Si-XM_004979921 (*Setaria italica*). The putative transmembrane (TM) domain and conserved acyltransferase (AT) domain are underlined

To elucidate the evolutionary relationship between ZmMs33 and its close homologues, the ZmMs33 protein sequence was used in BLAST_P_ queries in NCBI, MaizeGDB and RGAP databases. Meanwhile, we used AtATS1 and AtGPAT9 protein sequences to search for the putative *sn*-*1* GPATs in maize and rice. Consequently, there are 18 and 20 putative GPATs in the maize and rice genomes, respectively (Table S6). Because the GPAT family is relatively large, we only collected a total of 60 protein sequences, including all GPAT members from *Arabidopsis*, maize and rice, and the top 12 most closely related ZmMs33 homologues from other 12 plant species. We constructed a neighbour-joining phylogenetic tree of the 60 GPAT protein sequences, which are grouped into two main clades (Fig. 9). The *sn*-*1* clade included eight members that could be divided into two subclades: the plastidic AtATS1-related subclade and the ER-bound AtGPAT9-related subclade. The *sn*-*2* GPAT clade included all the other 52 GPAT members and was classified into two subclades. The first subclade was divided into three groups: the AtGPAT4/6/8-related group required for the biosynthesis of cutin, the AtGPAT5/7-related group associated with the biosynthesis of suberin, and the AtGPAT1-related group. The second subclade was related to AtGPAT2/3, which have been less functionally characterized. ZmMs33 was located to the monocot branch including OsGPAT3, a rice GPAT (LOC_Os12g37600), a maize GPAT (GRMZM2G033767) and eight other homologues in monocots such as *Oryza brachyantha* (Ob), *Sorghum bicolor* (Sb), *Triticum aestivum* (Ta), *Bradchypodium distachyon* (Bd), *Aegilops taushii (*Ats), *Dichanthelium oligosanthes* (Do), *Hordeum vulgare* (Hv) and *Setaria italica* (Si), whereas *Arabidopsis* AtGPAT2/3 occupied a relatively distant dicot branch with four homologues in dicots such as *Glycine max* (Gm), *Brassica napus* (Bn), *Raphanus sativus* (Rs) and *Brassica rapa* (Br) (Fig. 9). Based on the predicted amino acid sequence alignment, ZmMs33 shares 74, 69, 80 and 79% similarity with the orthologues of barley (AK376456), rice OsGPAT3 (Os11g45400), sorghum (XM_002449936), and millet (XM_004979921), respectively. Moreover, all the orthologues have a conserved AT domain at the same place (Fig. 10). Based on a BLAST_P_ search of the NCBI database, ZmMs33 was relatively close to OsGPAT3 (Table S3). Therefore, ZmMs33 is the orthologue of rice OsGPAT3, which may represent a unique *sn*-*2* GPAT specific to monocots.

## Discussion

### *ms33* is a complete male-sterile mutant and desirable for improving maize male-sterile line in hybrid seed production

In this study, we confirmed that *ms33* is a complete male-sterile mutant with no exerted anthers and no mature pollen grains (Figs. 1 and S2). Loss of *ZmMs33* expression in anthers (Fig. 8c) disrupts tapetum development and metabolism, which results in defective anther cuticle, blocked pollen exine formation, and eventually complete abortion of pollen grains (Figs. 2 and S3).The genetic analysis of the two F_2_ population derived from the crosses of *ms33*-*6029 *× Chang7-2 and *ms33*-*6038 *× Chang7-2 showed the 3:1 segregation ratio of the male-fertile to male-sterile plants, respectively (Table 1), which suggested both *ms33*-*6029* and *ms33*-*6038* were single recessive mutants. As the complete male-sterile phenotype of *ms33* mutants was genetically stable under different environments (Patterson 1995; Trimnell et al. 1999), *ms33* is a desirable genetic resource for improving maize male-sterile lines in hybrid seed production.

### Confirming gene function of *ZmMs33* based on targeted mutagenesis induced by a CRISPR/Cas9 system

*ms33* was narrowed down to an interval of 349 kb on chromosome 2L, and there were 15 putative genes in the interval (Fig. 3 and Table S3). To verify that *GRMZM2G070304* is the *ZmMs33* gene, two experiments were carried out: (1) functional complementation and (2) site-targeted mutagenesis using a CRISPR/Cas9 system. Transgenic lines with the coding sequence of *GRMZM2G070304* driven by its native promoter restored male fertility (Fig. 5). Based on targeted knockouts induced by the CRISPR/Cas9 system (Fig. 6), all the three types of knockout lines with different deletions in the *GRMZM2G070304* coding sequence showed a complete male-sterile phenotype (Fig. 7). These results demonstrate that *GRMZM2G070304* is responsible for restoring the male-sterile phenotype of the *ms33* mutants.

Recently, the CRISPR/Cas9 system has been widely used in gene function confirmation and genetic improvement in maize and other crops. A public-sector system (ISU Maize CRISPR) utilizing *Agrobacterium*-delivered CRISPR/Cas9 was developed for high-frequency targeted mutagenesis in four maize genes: *ZmAgo18a*, *ZmAgo18b, a1* and *a4* (Char et al. 2017). The CRISPR/Cas9 system was also successfully used for functional confirmation of *IPE1*, *APV1* and *ARGOS8* genes in maize (Chen et al. 2017; Shi et al. 2017; Somaratne et al. 2017). Furthermore, this system has been used in other crops such as rice (Li et al. 2016; Woo et al. 2015), wheat (Zhang et al. 2016), cotton (Wang et al. 2018), soybean (Cai et al. 2018) and *Camelina sativa* (Jiang et al. 2017).

In this study, the maize Hi-II hybrid line was transformed with three CRISPR/Cas9 vectors (Fig. 6) targeting different sites of endogenous *ZmMs33*. The three types of T_0_-generation maize plants homozygous for null alleles of *ZmMs33* were observed to be frame-shift mutations by 1-, 192- and 453-bp deletions (Fig. 7). All these site-targeted null mutant lines showed a completely male-sterile phenotype, indicating that both single gRNA/Cas9 and double gRNA/Cas9 constructs are highly efficient in producing site-specific gene mutants of *ZmMs33*. Therefore, it is feasible and efficient to use the CRISPR/Cas9 system for gene functional confirmation in maize.

### ZmMs33 and OsGPAT3 represent a monocot-specific *sn*-*2* GPAT family

*ZmMs33* encodes a GPAT protein with a conserved AT domain and shares high similarity with rice *OsGPAT3* (Men et al. 2017). Maize *ms33* and rice *osgpat3* mutants showed the similar male-sterile phenotypes, anther and pollen defects (Figs. 1, 2, S2 and S3). Moreover, similar with expression patterns of the rice *OsGPAT3* gene, *ZmMs33* was also expressed preferentially in maize immature anthers and root tissues (Fig. 8). Therefore, ZmMs33 appears to be an orthologue of OsGPAT3 and is required for male fertility in maize.

The land plant GPATs belong to a *sn*-2 GPAT family that differs from animal GPATs (Yang et al. 2012). The orthologues of ZmMs33 were found in 14 flowering plants, including 9 monocots and 5 dicots, and ZmMs33 and OsGPAT3 belong to a monocot-specific *sn*-*2* GPAT family, similar to the uncharacterized AtGPAT2 and AtGPAT3 in *Arabidopsis* (Fig. 9, Table S6). Although no enzyme activity was detected for AtGPAT2 and AtGPAT3, and their mutants showed no obvious phenotype in plant development (Yang et al. 2012), the *ms33* and *osgpat3* mutants displayed similar defective phenotypes in anther and complete male sterility (Men et al. 2017). We propose that ZmMs33 and OsGPAT3, together with their homologues in monocots, may have evolved divergently, leading to diversified functions different from those in dicots. Mutations in maize *ZmMs33* and rice *OsGPAT3* led to the same complete male-sterile phenotype, demonstrating that this kind of monocot-specific *sn*-*2* GPAT protein may be functionally conserved in monocot plants, where they play an indispensable role in male reproduction that is distinct from the role of their dicot counterparts. Therefore, these monocot-specific *sn*-*2 GPAT* genes may have potential application value in hybrid seed production in monocots.

### *ZmMs33* is the first *GPAT* gene identified in maize, and it controls male fertility through mediating glycerolipid synthesis

The tapetal lipid metabolism contributes significantly to anther wall cuticle and pollen exine formation (Shi et al. 2015). Glycerol is an essential backbone of plant polyesters such as suberin; however, its presence in plant cutin was discovered only comparatively recently (Graca et al. 2002; Pollard et al. 2008). GPATs play vital roles in mediating the initial step of glycerolipid synthesis pathway (Beisson et al. 2012; Yang et al. 2012).

In *Arabidopsis*, *AtGPAT1* is important for male fertility, and the *atgpat1* mutant exhibits a reduced ER stacks and mitochondrial dysfunction in the tapetum, thus leading to delayed programmed cell death of the tapetum cell layer and male semi-sterility. *AtGPAT6* has a similar expression pattern to *AtGPAT1* in anther development. In the *atgpat6* mutant, the ER system in the tapetum was severely impaired, ultimately resulting in defective pollen wall formation and male semi-sterility. The *atgpat1atgpat6* double mutant showed a defect of microspore release from tetrads and complete male sterility (Li et al. 2012; Zheng et al. 2003).

To date, there has been little gene isolation and characterization of GPATs in monocots. More recently, one member of the rice GPAT family, OsGPAT3, was found to be involved in the anther development and pollen formation in rice (Men et al. 2017). However, none of the *GPAT* genes in maize has been investigated until now. *ZmMs33* is the first *GPAT* gene identified in maize. Loss of *ZmMs33* expression in anthers disrupts anther development, leading to eventually complete abortion of pollen grains (Figs. 1, 2, S2, S3 and 8). Together with our data on ZmMs33 and the functions of OsGPAT3, AtGPAT1 and AtGPAT6, we concluded that these GPATs play important roles in anther development and male fertility through mediating glycerolipid synthesis in plants.

### The potential application of the *ZmMs33* gene in maize male sterile-line breeding and hybrid seed production

To date, hundreds of recessive genetic male-sterile mutants have been identified in plants, but their application in plant breeding and hybrid seed production has been limited because of the inability to propagate a pure male-sterile line via self-pollination. Recently, several strategies for maintaining and propagating male-sterile lines have been proposed based on the transgenic lines in plants such as maize (Wu et al. 2016; Zhang et al. 2018), rice (Chang et al. 2016), tobacco (Millwood et al. 2016) and Arabidopsis (Gils et al. 2008).

Most recently, to decrease the transgene transmission rate of the transgenic maintainer line through pollen, we developed a Multi-Control Sterility (MCS) system by transforming MCS constructs into the *ms7* mutant (Zhang et al. 2018). The MCS constructs contained five functional modules: (1) a male fertility gene *ZmMs7*, (2) two pollen-disrupted genes (*ZmAA* and *Dam*), (3) a screenable fluorescent colour marker gene (*DsRed2* or *mCherry*) and (4) an herbicide-resistant gene (*Bar*). The MCS constructs harbour five functional modules with the addition of *Bar* and *Dam* genes, which can ensure high purity of the male-sterile parent line by appropriate herbicide spraying of seedlings, and greatly decrease the transgene transmission rate as well as transgene flow risk through the use of two pollen-disrupting modules (Zhang et al. 2018). Therefore, the MCS system could be reconstructed based on the novel male-fertile *ZmMs33* gene and its mutants (*ms33*-*6029* and *ms33*-*6038*), and it is feasible to breed *ms33* male-sterile lines by combining traditional backcrossing and marker assisted selection (MAS) methods.

The orthologues of ZmMs33 were found in 14 flowering plants, and the GPAT family is divergent and has many members in major crops, including sorghum, barley and millet (Fig. 9), which have flowers that are not amenable to manual emasculation. Furthermore, ZmMs33, OsGPAT3 and their orthologues were found to be conserved in monocot plants and required for plant male gametogenesis (Fig. 10). Therefore, a reverse genetic approach could be adopted using targeted mutagenesis technologies like the CRISPR/Cas9 system (Kamthan et al. 2016; Zhang et al. 2016) and programmable DNA endonucleases (Cigan et al. 2017) or using chimeric repressor gene-silencing technology (Mitsuda et al. 2006) and RNAi (Fernández Gómez and Wilson 2014), which would lead to specific mutations in the corresponding orthologues of *ZmMs33* and produce male-sterile mutants in these crops. This will greatly enhance our understanding of the molecular mechanism of GPATs that are essential for male fertility in plants. In addition, the MCS system could be transferred into other major crops using artificial *ms* mutants and the corresponding fertility restoration genes, which will greatly expand the potential to produce male-sterile lines and hybrid seeds of important crops.

## Electronic supplementary material

Below is the link to the electronic supplementary material.

Supplementary material 1 (DOC 6456 kb)

## Abbreviations

AT: Acyltransferase
BLAST_P_: Basic Local Alignment Searching Tool-protein to protein
CD Search: Conserved domain search service
CMS: Cytoplasmic male sterility
CRISPR: Clustered regularly interspersed short palindromic repeats
CTAB: Cetyltrimethyl ammonium bromide
ER: Endoplasmic reticulum
GMS: Genetic male sterility
GPAT: Glycerol-3-phosphate acyltransferase
LPA: Lysophosphatidic acid
2-MAG: *sn*-*2* Monoacylglycerol
MaizeGDB: Maize Genetics and Genomics Database
MAS: Marker-assisted selection
MCS: Multi-control sterility
NCBI: National Center for Biotechnology Information
RGAP: Rice Genome Annotation Project
RT-PCR: Reverse transcription polymerase chain reaction
TM: Transmembrane

## References

[CR1] Albertsen MC, Phillips RL (1981). Developmental cytology of 13 genetic male sterile loci in maize. Can J Genet Cytol.

[CR2] Albertsen MC, Fox T, Trimnell M, Wu Y, Lowe L, Li B, Faller M (2009) *Msca1* nucleotide sequences impacting plant male fertility and method of using same. US patent US20090038027A1

[CR3] Albertsen M, Fox T, Leonard A, Li B, Loveland B, Trimnell M (2016) Cloning and use of the *ms9* gene from maize. US patent US20160024520A1

[CR4] Beisson F, Li-Beisson Y, Pollard M (2012). Solving the puzzles of cutin and suberin polymer biosynthesis. Curr Opin Plant Biol.

[CR5] Cai Y, Chen L, Liu X, Guo C, Sun S, Wu C, Jiang B, Han T, Hou W (2018). CRISPR/Cas9-mediated targeted mutagenesis of GmFT2a delays flowering time in soya bean. Plant Biotechnol J.

[CR6] Chang Z, Chen Z, Wang N, Xie G, Lu J, Yan W, Zhou J, Tang X, Deng XW (2016). Construction of a male sterility system for hybrid rice breeding and seed production using a nuclear male sterility gene. Proc Natl Acad Sci.

[CR7] Char SN, Neelakandan AK, Nahampun H, Frame B, Main M, Spalding MH, Becraft PW, Meyers BC, Walbot V, Wang K, Yang B (2017). An Agrobacterium-delivered CRISPR/Cas9 system for high-frequency targeted mutagenesis in maize. Plant Biotechnol J.

[CR8] Chen S, Songkumarn P, Liu J, Wang GL (2009). A versatile zero background T-vector system for gene cloning and functional genomics. Plant Physiol.

[CR9] Chen X, Zhang H, Sun H, Luo H, Zhao L, Dong Z, Yan S, Zhao C, Liu R, Xu C, Li S, Chen H, Jin W (2017). IRREGULAR POLLEN EXINE1 Is a novel factor in anther cuticle and pollen exine formation. Plant Physiol.

[CR10] Cigan AM, Unger E, Xu RJ, Kendall T, Fox TW (2001). Phenotypic complementation of *ms45* maize requires tapetal expression of MS45. Sex Plant Reprod.

[CR11] Cigan AM, Singh M, Benn G, Feigenbutz L, Kumar M, Cho MJ, Svitashev S, Young J (2017). Targeted mutagenesis of a conserved anther-expressed P450 gene confers male sterility in monocots. Plant Biotechnol J.

[CR12] Cui X, Wise RP, Schnable PS (1996). The rf2 nuclear restorer gene of male-sterile T-cytoplasm maize. Science.

[CR13] Djukanovic V, Smith J, Lowe K, Yang M, Gao H, Jones S, Nicholson MG, West A, Lape J, Bidney D, Carl Falco S, Jantz D, Alexander Lyznik L (2013). Male-sterile maize plants produced by targeted mutagenesis of the cytochrome P450-like gene (MS26) using a re-designed I-CreI homing endonuclease. Plant J.

[CR14] Eyster LA (1921). Heritable characters of maize. VII. Male sterile. J Hered.

[CR15] Feng Y, Zheng Q, Song H, Wang Y, Wang H, Jiang L, Yan J, Zheng Y, Yue B (2015). Multiple loci not only Rf3 involved in the restoration ability of pollen fertility, anther exsertion and pollen shedding to S type cytoplasmic male sterile in maize. Theor Appl Genet.

[CR16] Fernández Gómez J, Wilson ZA (2014). A barley PHD finger transcription factor that confers male sterility by affecting tapetal development. Plant Biotechnol J.

[CR17] Fox T, DeBruin J, Haug Collet K, Trimnell M, Clapp J, Leonard A, Li B, Scolaro E, Collinson S, Glassman K, Miller M, Schussler J, Dolan D, Liu L, Gho C, Albertsen M, Loussaert D, Shen B (2017). A single point mutation in Ms44 results in dominant male sterility and improves nitrogen use efficiency in maize. Plant Biotechnol J.

[CR18] Gils M, Marillonnet S, Werner S, Grutzner R, Giritch A, Engler C, Schachschneider R, Klimyuk V, Gleba Y (2008). A novel hybrid seed system for plants. Plant Biotechnol J.

[CR19] Gomez JF, Talle B, Wilson ZA (2015). Anther and pollen development: a conserved developmental pathway. J Integr Plant Biol.

[CR20] Graca J, Schreiber L, Rodrigues J, Pereira H (2002). Glycerol and glyceryl esters of omega-hydroxyacids in cutins. Phytochemistry.

[CR21] Hu YM, Tang JH, Yang H, Xie HL, Lu XM, Niu JH, Chen WC (2006). Identification and mapping of Rf-I an inhibitor of the Rf5 restorer gene for Cms-C in maize (*Zea mays* L.). Theor Appl Genet.

[CR22] Jiang WZ, Henry IM, Lynagh PG, Comai L, Cahoon EB, Weeks DP (2017). Significant enhancement of fatty acid composition in seeds of the allohexaploid, Camelina sativa, using CRISPR/Cas9 gene editing. Plant Biotechnol J.

[CR23] Jung KH, Han MJ, Lee YS, Kim YW, Hwang I, Kim MJ, Kim YK, Nahm BH, An G (2005). Rice Undeveloped Tapetum1 is a major regulator of early tapetum development. Plant Cell.

[CR24] Kamthan A, Chaudhuri A, Kamthan M, Datta A (2016). Genetically modified (GM) crops: milestones and new advances in crop improvement. Theor Appl Genet.

[CR25] Kelliher T, Walbot V (2012). Hypoxia triggers meiotic fate acquisition in maize. Science.

[CR26] Kohls S, Stamp P, Knaak C, Messmer R (2011). QTL involved in the partial restoration of male fertility of C-type cytoplasmic male sterility in maize. Theor Appl Genet.

[CR27] Kumar S, Stecher G, Tamura K (2016). MEGA7: molecular evolutionary genetics analysis version 7.0 for bigger datasets. Mol Biol Evol.

[CR28] Li Y, Beisson F, Koo AJ, Molina I, Pollard M, Ohlrogge J (2007). Identification of acyltransferases required for cutin biosynthesis and production of cutin with suberin-like monomers. Proc Natl Acad Sci USA.

[CR29] Li XC, Zhu J, Yang J, Zhang GR, Xing WF, Zhang S, Yang ZN (2012). Glycerol-3-phosphate acyltransferase 6 (GPAT6) is important for tapetum development in Arabidopsis and plays multiple roles in plant fertility. Mol Plant.

[CR30] Li J, Meng X, Zong Y, Chen K, Zhang H, Liu J, Li J, Gao C (2016). Gene replacements and insertions in rice by intron targeting using CRISPR-Cas9. Nat Plants.

[CR31] Ma J, Skibbe DS, Fernandes J, Walbot V (2008). Male reproductive development: gene expression profiling of maize anther and pollen ontogeny. Genome Biol.

[CR32] Men X, Shi J, Liang W, Zhang Q, Lian G, Quan S, Zhu L, Luo Z, Chen M, Zhang D (2017). Glycerol-3-Phosphate Acyltransferase 3 (OsGPAT3) is required for anther development and male fertility in rice. J Exp Bot.

[CR33] Millwood RJ, Moon HS, Poovaiah CR, Muthukumar B, Rice JH, Abercrombie JM, Abercrombie LL, Green WD, Stewart CN (2016). Engineered selective plant male sterility through pollen-specific expression of the EcoRI restriction endonuclease. Plant Biotechnol J.

[CR34] Mitsuda N, Hiratsu K, Todaka D, Nakashima K, Yamaguchi-Shinozaki K, Ohme-Takagi M (2006). Efficient production of male and female sterile plants by expression of a chimeric repressor in Arabidopsis and rice. Plant Biotechnol J.

[CR35] Moon J, Skibbe D, Timofejeva L, Wang CJ, Kelliher T, Kremling K, Walbot V, Cande WZ (2013). Regulation of cell divisions and differentiation by MALE STERILITY32 is required for anther development in maize. Plant J.

[CR36] Nan GL, Zhai J, Arikit S, Morrow D, Fernandes J, Mai L, Nguyen N, Meyers BC, Walbot V (2017). MS23, a master basic helix-loop-helix factor, regulates the specification and development of the tapetum in maize. Development.

[CR37] Nishida I, Tasaka Y, Shiraishi H, Murata N (1993). The gene and the RNA for the precursor to the plastid-located glycerol-3-phosphate acyltransferase of *Arabidopsis thaliana*. Plant Mol Biol.

[CR38] Patterson E (1995). Tests of male sterile mutants. Maize Genet Coop Newslett.

[CR39] Petit J, Bres C, Mauxion JP, Tai FW, Martin LB, Fich EA, Joubes J, Rose JK, Domergue F, Rothan C (2016). The glycerol-3-phosphate acyltransferase GPAT6 from tomato plays a central role in fruit cutin biosynthesis. Plant Physiol.

[CR40] Pollard M, Beisson F, Li Y, Ohlrogge JB (2008). Building lipid barriers: biosynthesis of cutin and suberin. Trends Plant Sci.

[CR41] Shi J, Cui M, Yang L, Kim YJ, Zhang D (2015). Genetic and biochemical mechanisms of pollen wall development. Trends Plant Sci.

[CR42] Shi J, Gao H, Wang H, Lafitte HR, Archibald RL, Yang M, Hakimi SM, Mo H, Habben JE (2017). ARGOS8 variants generated by CRISPR-Cas9 improve maize grain yield under field drought stress conditions. Plant Biotechnol J.

[CR43] Shockey J, Regmi A, Cotton K, Adhikari N, Browse J, Bates PD (2016). Identification of Arabidopsis GPAT9 (At5g60620) as an essential gene involved in triacylglycerol biosynthesis. Plant Physiol.

[CR44] Skibbe DS, Fernandes JF, Medzihradszky KF, Burlingame AL, Walbot V (2009). Mutator transposon activity reprograms the transcriptomes and proteomes of developing maize anthers. Plant J.

[CR45] Somaratne Y, Tian Y, Zhang H, Wang M, Huo Y, Cao F, Zhao L, Chen H (2017). ABNORMAL POLLEN VACUOLATION1 (APV1) is required for male fertility by contributing to anther cuticle and pollen exine formation in maize. Plant J.

[CR46] Tan Y, Li S, Xie H, Duan S, Wang T, Zhu Y (2011). Genetical and molecular analysis reveals a cooperating relationship between cytoplasmic male sterility- and fertility restoration-related genes in Oryza species. Theor Appl Genet.

[CR47] Tang JH, Fu ZY, Hu YM, Li JS, Sun LL, Ji HQ (2006). Genetic analyses and mapping of a new thermo-sensitive genic male sterile gene in maize. Theor Appl Genet.

[CR48] Timofejeva L, Skibbe DS, Lee S, Golubovskaya I, Wang R, Harper L, Walbot V, Cande WZ (2013). Cytological characterization and allelism testing of anther developmental mutants identified in a screen of maize male sterile lines. G3 (Bethesda).

[CR49] Trimnell MR, Patterson E, Fox TW, Bedinger P, Albertsen MC (1999). New chromosome 2L male-sterile mutant ms33 and alleles. Maize Genet Coop Newslett.

[CR50] Vernoud V, Laigle G, Rozier F, Meeley RB, Perez P, Rogowsky PM (2009). The HD-ZIP IV transcription factor OCL4 is necessary for trichome patterning and anther development in maize. Plant J.

[CR51] Wan XY, Wan JM, Jiang L, Wang JK, Zhai HQ, Weng JF, Wang HL, Lei CL, Wang JL, Zhang X, Cheng ZJ, Guo XP (2006). QTL analysis for rice grain length and fine mapping of an identified QTL with stable and major effects. Theor Appl Genet.

[CR52] Wan X, Weng J, Zhai H, Wang J, Lei C, Liu X, Guo T, Jiang L, Su N, Wan J (2008). Quantitative trait loci (QTL) analysis for rice grain width and fine mapping of an identified QTL allele gw-5 in a recombination hotspot region on chromosome 5. Genetics.

[CR53] Wang CJ, Nan GL, Kelliher T, Timofejeva L, Vernoud V, Golubovskaya IN, Harper L, Egger R, Walbot V, Cande WZ (2012). Maize multiple archesporial cells 1 (mac1), an ortholog of rice TDL1A, modulates cell proliferation and identity in early anther development. Development.

[CR54] Wang DX, Skibbe DS, Walbot V (2013). Maize *Male sterile 8 (Ms8)*, a putative β-1,3-galactosyltransferase, modulates cell division, expansion, and differentiation during early maize anther development. Plant Reprod.

[CR55] Wang P, Zhang J, Sun L, Ma Y, Xu J, Liang S, Deng J, Tan J, Zhang Q, Tu L, Daniell H, Jin S, Zhang X (2018). High efficient multisites genome editing in allotetraploid cotton (Gossypium hirsutum) using CRISPR/Cas9 system. Plant Biotechnol J.

[CR56] Williams ME (1995). Genetic engineering for pollination control. Trends Biotech.

[CR57] Woo JW, Kim J, Kwon SI, Corvalan C, Cho SW, Kim H, Kim SG, Kim ST, Choe S, Kim JS (2015). DNA-free genome editing in plants with preassembled CRISPR-Cas9 ribonucleoproteins. Nat Biotechnol.

[CR58] Wu Y, Fox TW, Trimnell MR, Wang L, Xu RJ, Cigan AM, Huffman GA, Garnaat CW, Hershey H, Albertsen MC (2016). Development of a novel recessive genetic male sterility system for hybrid seed production in maize and other cross-pollinating crops. Plant Biotechnol J.

[CR59] Xing HL, Dong L, Wang ZP, Zhang HY, Han CY, Liu B, Wang XC, Chen QJ (2014). A CRISPR/Cas9 toolkit for multiplex genome editing in plants. BMC Plant Biol.

[CR60] Yang W, Simpson JP, Li-Beisson Y, Beisson F, Pollard M, Ohlrogge JB (2012). A land-plant-specific glycerol-3-phosphate acyltransferase family in Arabidopsis: substrate specificity, *sn*-*2* preference, and evolution. Plant Physiol.

[CR61] Zhang Y, Liang Z, Zong Y, Wang Y, Liu J, Chen K, Qiu JL, Gao C (2016). Efficient and transgene-free genome editing in wheat through transient expression of CRISPR/Cas9 DNA or RNA. Nat Commun.

[CR62] Zhang D, Wu S, An X, Xie K, Dong Z, Zhou Y, Xu L, Fang W, Liu S, Liu S, Zhu T, Li J, Rao L, Zhao J, Wan X (2018). Construction of a multi-control sterility system for a maize male-sterile line and hybrid seed production based on the ZmMs7 gene encoding a PHD-finger transcription factor. Plant Biotechnol J.

[CR63] Zheng Z, Xia Q, Dauk M, Shen W, Selvaraj G, Zou J (2003). Arabidopsis AtGPAT1, a member of the membrane-bound glycerol-3-phosphate acyltransferase gene family, is essential for tapetum differentiation and male fertility. Plant Cell.

[CR64] Zhu SQ, Zhao H, Zhou R, Ji BH, Dan XY (2009). Substrate selectivity of glycerol-3-phosphate acyl transferase in rice. J Integr Plant Biol.

