# Supplementary material for: Map-based cloning and characterization of Zea mays male sterility33 (ZmMs33) gene, encoding a glycerol-3-phosphate acyltransferase
Source: Theor Appl Genet. 2018 Mar 15;131(6):1363–78. doi: 10.1007/s00122-018-3083-9 (PMC5945757; doi:10.1007/s00122-018-3083-9)
Supplement: Supplementary file 1 — Supplementary material 1 (DOC 6456 kb) [file 122_2018_3083_MOESM1_ESM.doc]

# Supporting information

# Map-based cloning and characterization of *Zea mays male sterility33* (*ZmMs33*) gene, encoding a glycerol-3-phosphate acyltransferase

Ke Xie^1,2#^, Suowei Wu^1,2#^, Ziwen Li^1,2#^, Yan Zhou^2^, Danfeng Zhang^2^, Zhenying Dong^1^, Xueli An^1,2^, Taotao Zhu^1^, Simiao Zhang^1^, Shuangshuang Liu^2^, Jinping Li^2^, Xiangyuan Wan^1,2^*

**Fig. S1** Activities of glycerol-3-phosphate acyltransferase (GPAT). LPA, lysophosphatidic acid; MAG, monoacylglycerol. The schematic diagram was reproduced and modified according to the reference (Beisson et al. 2012).

**
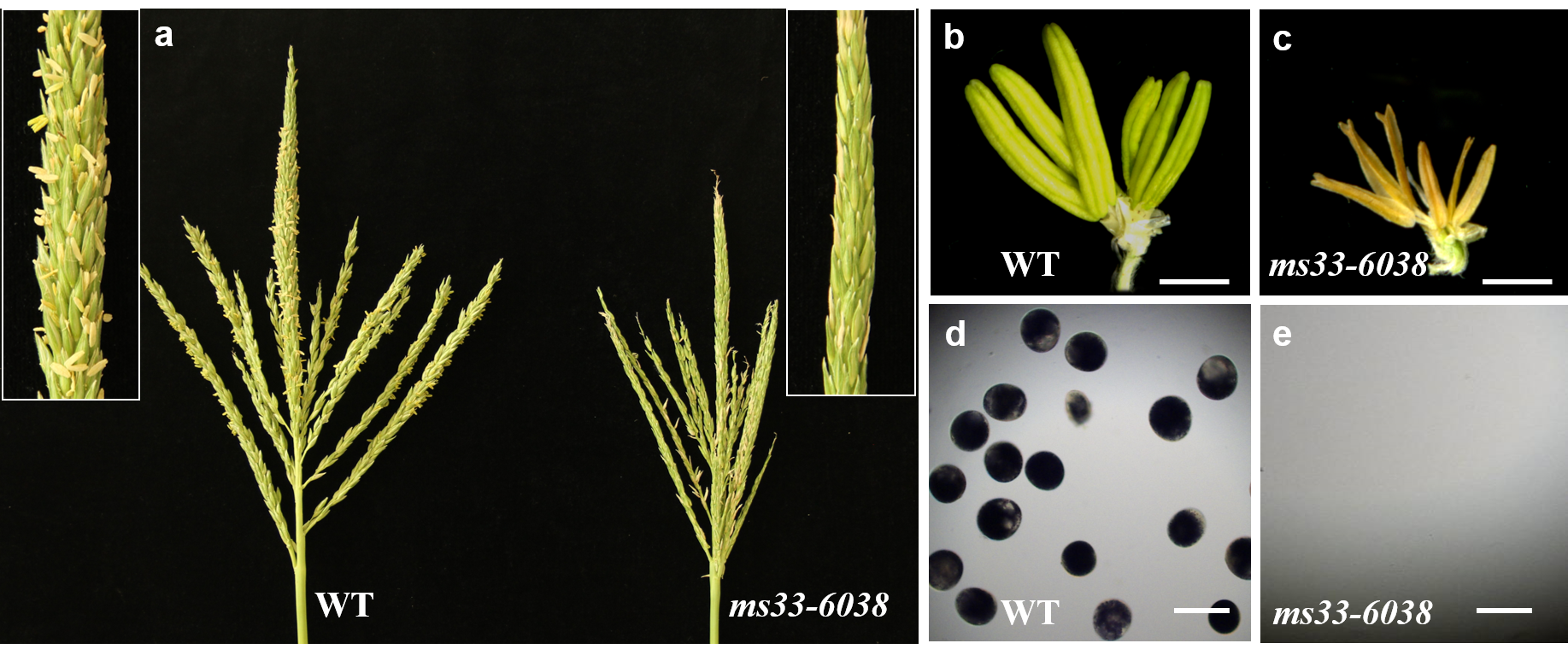
**

**Fig. S2** Phenotypic comparison of the wild type (WT) and *ms33-6038* mutant maize. **a** The tassel of the WT (left) and *ms33-6038* (right). **b** and **c** The spikelet of WT and *ms33-6038* with the glume, lemma and palea removed and showing the two florets with three anthers each. **d** and **e** The pollen of WT and *ms33-6038* stained with I_2_-KI. Bars = 2 mm (b, c), 100μm (d, e).

**Fig. S3** Appearance of the anther and pollen grain in wild-type (WT) and *ms33-6038* mutant maize under scanning electron microscopy. **a** WT and **b** *ms33-6038* anthers at stage 13 of development. **c, c1** and **d** Pollen grains of WT (c and c1) and no pollen grains in *ms33-6038* (d) at stage 12. **e, f** The outermost surface of the epidermis of WT (e) and *ms33-6038* (f) anthers at stage 12. **g, h** The inner surface of the anther wall layers of WT (g) and *ms33-6038* (h) anthers at stage 12. Bars=1 mm (a, b), 200 μm (c), 15 μm (c1), 0.5 mm (d), 10 μm (e, f), 5 μm (g, h).

**Fig. S4** Sequencing and nucleotide alignment of ZmMs33 RT-PCR products in maize immature anther and root tissue. ZmMs33-CDS, the coding DNA sequence of ZmMs33; Ms33-RT-PCR-A and Ms33-RT-PCR-R, the sequence of RT-PCR products of ZmMs33 in anther (A) and root (R), respectively. The primer sequences of Ms33-RT were indicated by underline.


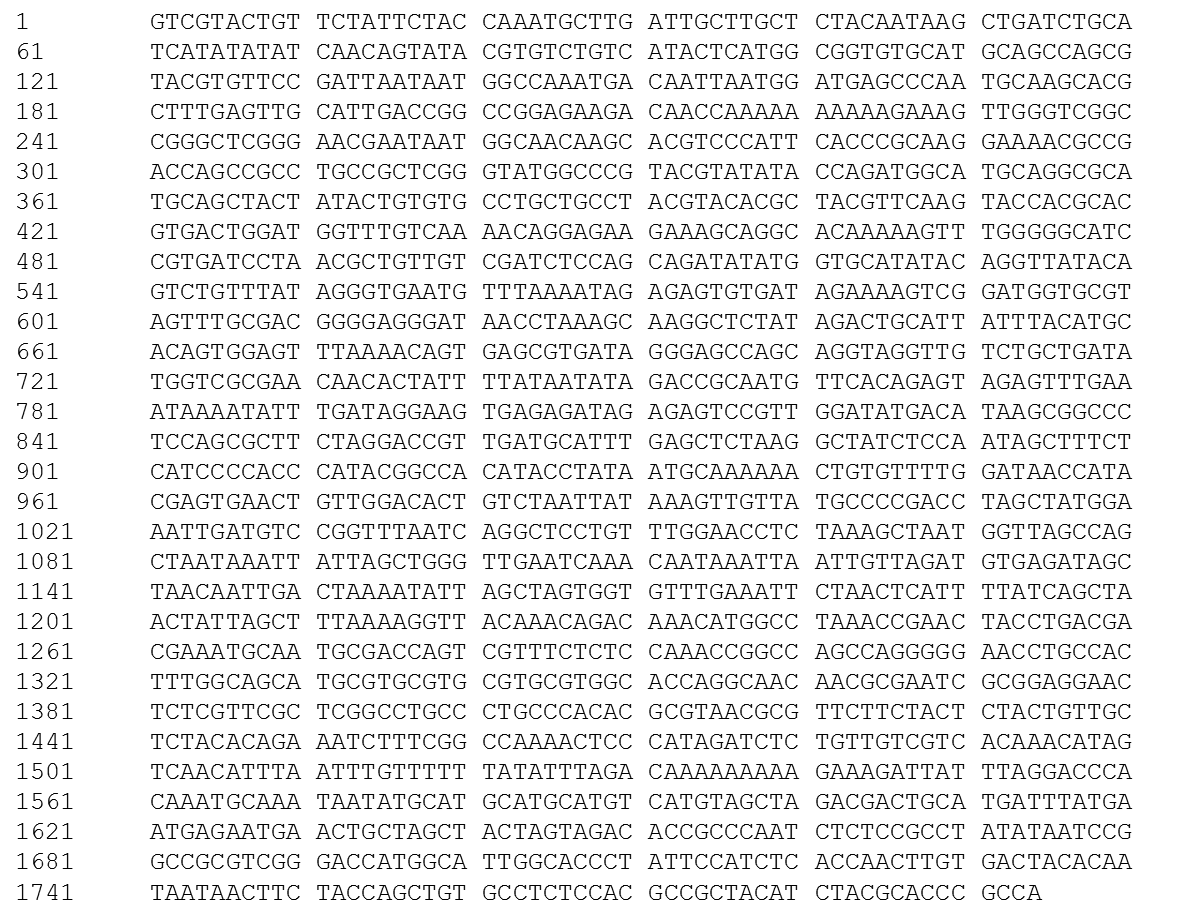


Fig. S5 The DNA sequence information of *ZmMs33* (*GRMZM2G070304*) full-length promoter.


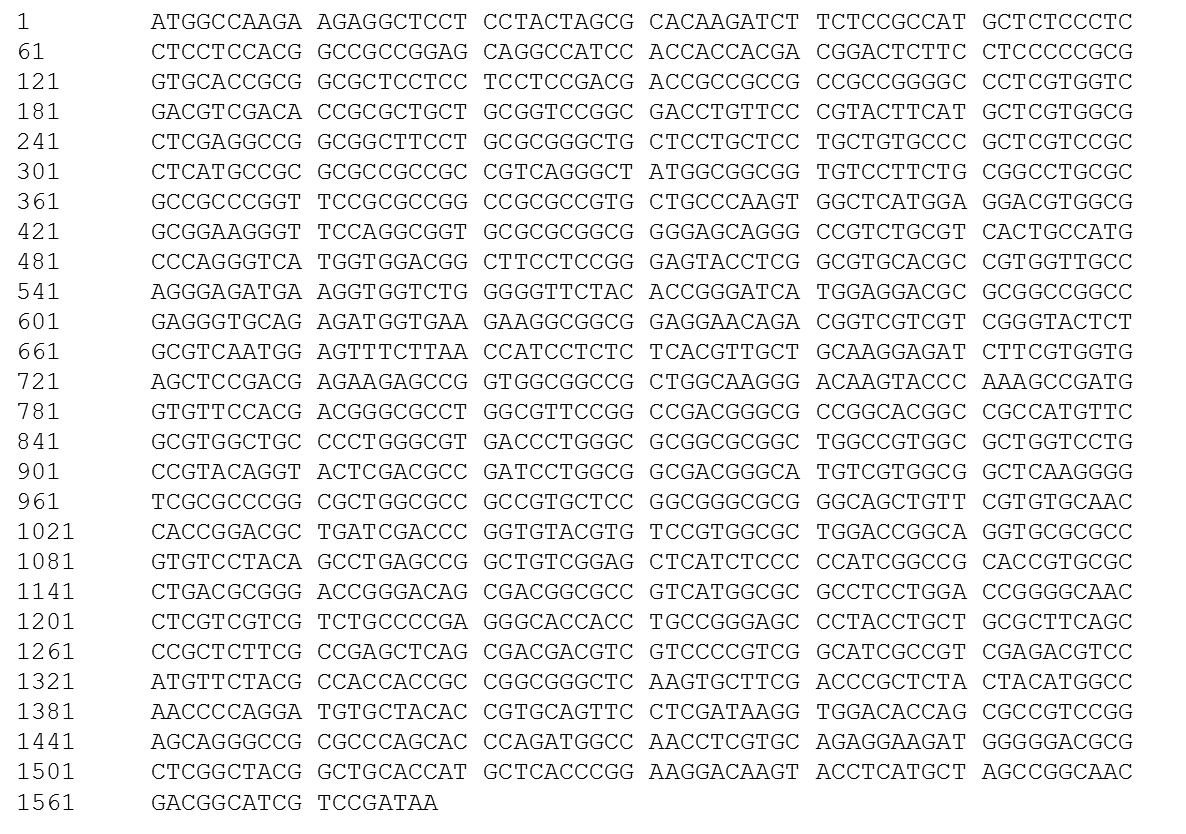


Fig. S6 The coding DNA sequence information of *ZmMs33* (*GRMZM2G070304*) gene used for genetic complementation. The 1578-bp cDNA sequence encodes a 525-amino acid protein in maize.


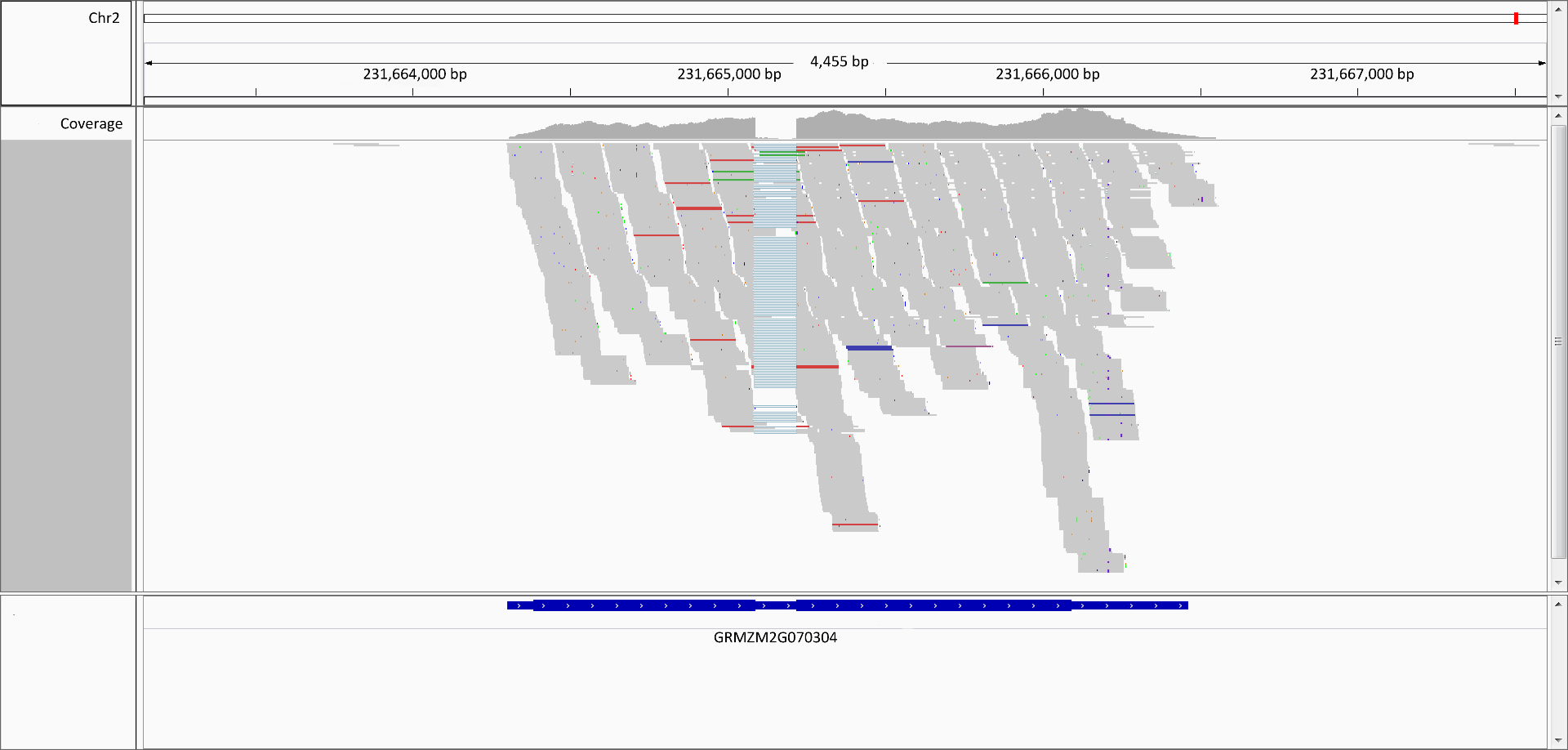

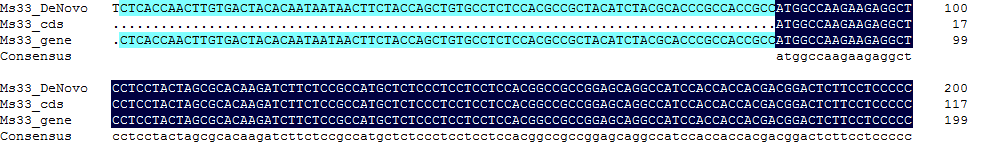


a

b

Fig. S7 The RNA-seq data of *ZmMs33* (*GRMZM2G070304*) in immature anthers of the *ZmMs33/ZmMs33* fertile plants. **a** The RNA-seq reads of *ZmMs33* (*GRMZM2G070304*). **b** The alignment of the RNA-seq data (*Ms33*_DeNovo), *Ms33* coding DNA sequence (*Ms33*_cds) and *Ms33* genomic DNA sequence (*Ms33*_gene) in the 5’ end. The sequence alignment was performed by DNAMAN.

Fig. S8 Mutation analysis of the three types of knockout lines generated by a CRISPR/Cas9 system. **a** *Ms33-Cas9-1*, **b** *Ms33-Cas9-2*, **c** *Ms33-Cas9-3*. All the mutations induced by CRISPR/Cas9 were detected by direct sequencing of the PCR products. The inserted (+) or deleted (−) nucleotides are denoted. Sequences framed in red are the sgRNA targets in the *Ms33* gene locus. WT indicates the un-edited sequence of *Ms33* target gene. The sequence alignment was performed by DNAMAN. Dots, deleted bases. The type of indel and the number of indels of the same type are indicated in the right.

**Table S1 The molecular marker information used for *ZmMs33* gene mapping**

| **Primers** | **Forward（5’-3’）** | **Reverse（5’-3’）** | |
| --- | --- | --- | --- |
| mmc0381 | GTGGCCCTGTTGATGAG | CGACGAGTACCAGGCAT |  |
| bnlg1940 | CCTTTTGTTTCAGGCCGTTA | CAGCAGCCTGATGATGAACA |  |
| umc1230 | GTACGACCGTTGAAACTGTTGTTTT | GCGATTTCAACTATTTGTGGTAAAGG |  |
| umc1551 | CACCGGAACACCTTCTTACAGTTT | CGAAACCTTCTCGTGATGAGC |  |
| umc2214 | CTGGATGAGGAGGAAGAATACGAG | ACCCCCTGATTCTCTCTTACGTTT |  |
| umc1252 | GCGTCGGAGAAGTACATCAAGTTT | CTTCTGCATCATCATCATCGTCTT |  |
| bnlg1893 | AATCCTGTAGCGTGTGTCCC | TAACTGAGTTGTTGAAGGAAATTG |  |
| EP95 | AACCTAGCAGTGGTCGTTGG | GACGTAGTTCTGTCCAGCCC |  |
| EP97 | CGAGATGACGTTGAAACACG | TGTGAAAGTTGGCATTGACC |  |
| EP198 | AGCCTCGATTCCTTCATCCG | GCAGTAAAGCCACTACAACAGG |  |
| EP603 | GAAGCACTCAATCGAGCC | GCATAGAAGCCCTTACACAC |  |
| EP605 | GGCACTCATGGTCAACA | ACTCGAAATTCAGATTGCAGG |  |
| EP480 | CAACAAAGCTAGGATTCCTCGAT | ACAGCCCCTTGAGGTCCA |  |

**Table S2 PCR primers used in this study**

| Primer | Forward (5'-3') | Reverse (5'-3') | Purpose |
| --- | --- | --- | --- |
| Ms33-FL | GGGATAACCTAAAGCAAGGC | GCACCGCAGAGATACAATAAAG | *ZmMs33* full length gDNA |
| Ms33-FC | CTCACCAACTTGTGACTACAC | GCACCGCAGAGATACAATAAAG | *ZmMs33* full length cDNA |
| Ms33-ID | GGCCACAAGCTGCTCAACCT | CTACCTTCCTTGCAATGGATAAC | Identification of *ms33-6029* |
| Ms33-RT | GCAGAGATGGTGAAGAAGGC | ACACCATCGGCTTTGGGTA | RT-PCR of *ZmMs33* |
| ACTIN-RT | GGCCACAAGCTGCTCAACCT | ATGTGGTTGCCCAGGGACTT | RT-PCR of *ZmACTIN* |
| ZmGAPDH | CAACGACCCCTTCATCACCACG | ATACTCAGCGCCAGCCTCACCC | RT-PCR of *ZmGAPDH* |
| Ms33-ProP | CAGAAGCTTCGTCGTACTGTTCTATTCTACC | CTTCTTGGCCATGGcctaGGTGGCGGGTGCGTA | Vector, p*ZmMs33pro::ZmMs33* |
| Ms33-CDSP | CCCGCCACCTAGGCCATGGCCAAGAAGAGGCTC | CACGGATCCTTATCGGACGATGCCGTCGTTG | Vector, p*ZmMs33pro::ZmMs33* |
| Cas9-2g-1-T1 | ATATATGGTCTCTGGCGGAAGATCTTGTGCGCTAGT | GGAAGATCTTGTGCGCTAGTGTTTTAGAGCTAGAAATAGC | Vector, pCas9-2g-1 |
| Cas9-2g-1-T2 | AACGTTCCCGTACTTCATGCTCCGCTTCTTGGTGCC | ATTATTGGTCTCTAAACGTTCCCGTACTTCATGCTCC | Vector, pCas9-2g-1 |
| Cas9-2g-2-T1 | ATATATGGTCTCTGGCGTTCTACACCGGGATCATGG | GTTCTACACCGGGATCATGGGTTTTAGAGCTAGAAATAGC | Vector, pCas9-2g-2 |
| Cas9-2g-2-T2 | AACGCCGTACAGGTACTCGACGCGCTTCTTGGTGCC | ATTATTGGTCTCTAAACGCCGTACAGGTACTCGACGC | Vector, pCas9-2g-2 |
| Cas9-1g-1 | GGCGAGTAGGAGGAGCCTCTTCT | AAACAGAAGAGGCTCCTCCTACT | Vector, pCas9-1g-1 |
| Bar-P | TCTACCATGAGCCCAGAAC | TCAAATCTCGGTGACGGGCA | Transgene determination |

**Table S3 The putative 15 gene models in the *ZmMs33* fine-mapping interval**

| **Gene models** | **Arabidopsis best hit** | **Rice best hit** | **Description** |
| --- | --- | --- | --- |
| GRMZM2G372180 | ATK4 AT5G27000.1 | LOC_Os11g44880.1 | kinesin |
| GRMZM2G070371 | AT2G37650.1 | LOC_Os11g47870.1 | GRAS family transcription factor |
| GRMZM2G070304 | ATGPAT3 AT4G01950.1 | LOC_Os11g45400.1 | glycerol-3-phosphate acyltransferase |
| GRMZM2G107501 | AT5G41460.1 | LOC_Os04g48950.1 | DUF，fringe-related protein |
| GRMZM2G107508 | no hit | no hit |  |
| GRMZM2G003182 | AT1G08540.1 | LOC_Os03g16430.1 | RNApolymerase sigma subunit |
| GRMZM5G820632 | no hit | no hit |  |
| GRMZM2G180172 | LCD1 AT2G37860.3 | LOC_Os07g13590.1 | DUF3411 |
| GRMZM2G156091 | no hit | no hit |  |
| GRMZM2G156127 | AT3G55700.1 | LOC_Os03g60960.1 | Glycosyltransferase superfamily protein |
| GRMZM2G576456 | no hit | no hit |  |
| GRMZM2G471160 | AT3G46670.1 (UGT76E11) | LOC_Os07g13770.1 | UDP-glucosyl transferase |
| AC148152.3_FG008 | AT3G06510.1 (ATSFR2, SFR2) | LOC_Os11g45710.1 | Glycosyl hydrolase superfamily protein |
| AC148152.3_FG007 | no hit | no hit |  |
| AC148152.3_FG006 | AT5G13930.1(ATCHS,CHS, TT4) | LOC_Os07g17010.1 | Chalcone and stilbene synthase family protein |

**Table S4 Transgenic complementation of *ms33-6029* with pMs33-Ms33 vector**

| Number of independent transformants | Copy number of T-DNA insertion | | Phenotype of transgenic plants  (pMs33-Ms33/*ms33-6029*) | |
| --- | --- | --- | --- | --- |
|  | single | 2-3 | male fertile | male sterile |
| 15 | 9 | 6 | 15 | 0 |

**Table S5** **Targeted knockout of ZmMs33 in maize using a CRISPR/cas9 system**

| Constructs of CRISPR/Cas9 | Number of independent transformants | Phenotype of transgenic plants (T_0_) | |
| --- | --- | --- | --- |
|  |  | male fertile | male sterile |
| pCas9-1g | 17 | 0 | 17 |
| pCas9-2g-1 | 6 | 0 | 6 |
| pCas9-2g-2 | 13 | 0 | 13 |

**Table S6 The putative GPAT family members in five model plants**

Note：BLASTp in NCBI, RGAP and MaizeGDB databases using ZmMs33, AtATS1 and AtGPAT9 protein sequences.
